# Epidemiology and Spectrum of Imported Infectious Diseases in Children and Adolescents Returning to Europe: A Systematic Review

**DOI:** 10.3390/pathogens15060621

**Published:** 2026-06-09

**Authors:** Jakub Niestępski, Jakub Marek Baran, Zuzanna Waszak, Joanna Jarzębska, Damian Grusiecki, Maja Śmigielska, Magdalena Marczyńska, Maria Pokorska-Śpiewak

**Affiliations:** 1 Department of Pediatric Infectious Diseases, Hospital for Infectious Diseases in Warsaw, 01-201 Warsaw, Poland; jak.baran00@gmail.com (J.M.B.); magdalena.marczynska@wum.edu.pl (M.M.); maria.pokorska-spiewak@wum.edu.pl (M.P.-Ś.); 2 Department of Children’s Infectious Diseases, Medical University of Warsaw, 01-201 Warsaw, Poland

**Keywords:** imported infectious diseases, pediatric travelers, Europe, malaria, dengue, leishmaniasis, schistosomiasis, travel medicine, visiting friends and relatives (VFR) travelers, epidemiology

## Abstract

**Background:** International travel and global mobility have led to an increasing number of pediatric patients presenting with infections acquired outside Europe. However, epidemiological data on imported infectious diseases in children remain fragmented, with substantial heterogeneity across studies. This systematic review aimed to synthesize current evidence on the spectrum, distribution, and epidemiological characteristics of imported infections in children and adolescents returning to Europe. **Methods:** A systematic review was conducted in accordance with PRISMA 2020 guidelines and registered in PROSPERO (CRD420251245531). PubMed, Scopus, and the Cochrane Library were searched for studies published between 2010 and December 2025. Studies reporting laboratory-confirmed or clinically diagnosed imported infections in pediatric populations (0–18 years) returning to Europe were included. Data were extracted and synthesized descriptively, with separate analyses for malaria-specific, non-malarial, and syndromic cohorts. **Results:** A total of 31 studies was included. Malaria was the most consistently reported infection, predominantly caused by *Plasmodium falciparum* and most frequently reported in association with travel to sub-Saharan Africa, which accounted for the largest proportion of reported exposures in clinical cohorts. Visiting friends and relatives (VFR) was the dominant travel category, representing between 45.8% and 87.4% of cases in disaggregated malaria cohorts and a substantial proportion across non-malarial studies. Non-malarial infections—including gastrointestinal, bacterial, parasitic, and viral diseases—represented a substantial proportion of cases in mixed and post-travel cohorts, with gastrointestinal illness frequently constituting the leading diagnosis. Considerable heterogeneity was observed across studies in terms of design, diagnostic approaches, and reporting practices, precluding formal meta-analysis. **Conclusions:** Imported infections in pediatric travelers encompass a broad and heterogeneous spectrum extending beyond malaria alone. Diagnostic approaches should integrate travel history, geographic exposure, and clinical presentation. Standardized pediatric-specific surveillance and harmonized reporting are needed to improve comparability and support more accurate epidemiological assessment.

## 1. Introduction

International travel and global mobility have increased significantly in recent decades, resulting in a growing number of children and adolescents being exposed to infectious agents that are uncommon or non-endemic in Europe. Global tourism has reached unprecedented levels, with international travel involving a substantial proportion of the pediatric population [1]. This expansion in travel has been accompanied by an increase in travel-associated infectious diseases, particularly among individuals returning from tropical and subtropical regions [2].

Children represent a distinct epidemiological group in the population, and their exposure to infectious diseases is characterized by age-specific, incomplete immunization status, and specific behavioral factors. Available data suggest that pediatric travelers are more likely to present with febrile illnesses, gastrointestinal diseases, and vector-borne diseases associated with international travel [3,4]. Malaria, dengue fever, enteric fever, and parasitic diseases such as schistosomiasis and leishmaniasis are the most commonly reported imported diseases in pediatric travelers [3,4,5].

The epidemiology of imported infectious diseases in pediatric travelers is influenced by a number of factors, including the destination region, duration of stay, environmental exposure, and purpose of travel. In particular, children traveling to visit friends and relatives (VFR travelers) are consistently overrepresented among reported cases due to the lower uptake of pre-travel medical advice and increased exposure to local transmission settings [6]. Additionally, climatic conditions and seasonal variations in vector activity further influence the risk of infection and pathogen distribution [7].

The clinical presentation of imported infections in the pediatric population is often nonspecific, with symptoms such as fever, diarrhea, and malaise overlapping with those of common childhood illnesses. This frequently results in diagnostic delays, particularly in non-endemic European settings where clinician familiarity with tropical diseases may be limited [4,8]. Moreover, the incubation period of many travel-related infections may extend beyond the return date, complicating the identification of travel-associated etiology [9].

Imported infections in children are associated with substantial healthcare utilization, including frequent hospital admissions for diagnostic evaluation and management. Although mortality remains relatively low, severe disease courses and complications, particularly in malaria and other infections requiring delayed recognition or hospital-based management, have been reported [3,10]. These observations underscore the clinical and public health importance of travel-associated infections in the pediatric population.

Despite the growing body of research in travel medicine, epidemiological evidence specifically focusing on children and adolescents remains fragmented. Many studies either combine adult and pediatric populations without stratified analyses or focus on single pathogens, limiting the ability to draw comprehensive conclusions [8]. Furthermore, heterogeneity in study design, surveillance systems, and reporting standards across European countries complicates direct comparisons and the synthesis of available data.

Therefore, a structured systematic synthesis of current evidence is needed to better characterize the reported frequency, spectrum, and epidemiological characteristics of imported infectious diseases in pediatric travelers. The aim of this systematic review is to evaluate the epidemiology, clinical spectrum, and epidemiological correlates of laboratory-confirmed or clinically diagnosed imported infections in children and adolescents (0–18 years) returning to Europe after international travel, with particular emphasis on infections acquired in endemic regions.

## 2. Materials and Methods

### 2.1. Study Design and Reporting Framework

This study was conducted as a systematic review of imported infectious diseases in children and adolescents returning to Europe. The review was designed and reported in accordance with the PRISMA 2020 framework and was based on a prospectively developed protocol registered in PROSPERO (CRD420251245531) (Supplementary File S1). Given the heterogeneity of the available evidence, the review combined a systematic study identification process with a structured descriptive synthesis focused on clinically relevant epidemiological patterns.

### 2.2. Conceptual Scope of the Review

The review was designed to examine imported infections as a clinical and epidemiological entity in pediatric post-travel populations. For the purposes of this review, an imported infection was defined as any infection acquired outside Europe and diagnosed after return, regardless of whether the causative pathogen is endemic within Europe. This definition was chosen to reflect real-world pediatric travel medicine, in which the diagnostic challenge is determined primarily by the place of acquisition and travel history rather than by whether the pathogen is strictly non-endemic in the receiving country.

For the purposes of this review, ‘Europe’ was operationally defined as the European Union and its 27 current member states, as specified in the geographic search terms documented in Supplementary Table S1. One included study, by Bird et al. [11], was conducted across pediatric emergency departments in both the United Kingdom and the Republic of Ireland (PERUKI network) and was retained on the basis of its Irish component, which falls within the defined geographic scope.

Accordingly, the review did not aim to provide an exhaustive inventory of all infectious diseases that may be imported into Europe. Instead, it was intended to characterize the epidemiology of those infections that were most consistently reported and clinically relevant within pediatric post-travel cohorts. This distinction is important because the published literature on imported infections is not organized as a comprehensive catalog of all possible travel-associated pathogens. Rather, it is shaped by clinical presentation, healthcare setting, surveillance priorities, and study design. As a result, the final spectrum of infections represented in the review reflects both the predefined review scope and the reporting structure of the available evidence.

### 2.3. Eligibility Criteria

Eligible studies reported imported infectious diseases in children or adolescents aged 0–18 years returning to Europe after international travel and provided extractable pediatric clinical or epidemiological data. Both disease-specific studies, such as malaria-focused cohorts and broader post-travel cohorts, including febrile illness and general imported infection studies, were eligible, provided that pediatric results could be identified and interpreted. Studies with slightly broader pediatric age definitions, such as <20 years, were included when they predominantly represented children and adolescents and did not materially affect the interpretation of pediatric epidemiological patterns.

Studies were excluded if they focused exclusively on adults, did not provide pediatric-specific data, lacked extractable numerical information, or concerned infections acquired within Europe without a clear travel-associated exposure. Reviews, editorials, single case reports, conference abstracts, and other non-primary study designs were also excluded.

### 2.4. Rationale for the Disease Scope

The disease scope was intentionally focused rather than exhaustive. The review prioritized infections that fulfilled at least one of the following criteria within pediatric post-travel cohorts: they were frequently reported, had clear epidemiological relevance in returning children, represented an important diagnostic consideration in clinical practice, or were sufficiently consistently described to allow meaningful synthesis across studies. This included malaria, dengue, leishmaniasis, schistosomiasis, typhoid and paratyphoid fever, chikungunya, Zika virus infection, and travel-associated enteric infections, including pathogen-defined bacterial and parasitic gastrointestinal infections. Conditions were included if they met at least one of three predefined criteria: consistent reporting in pediatric post-travel cohorts from European settings; established epidemiological relevance as causes of febrile or systemic illness in returning children; or sufficient case volume for meaningful descriptive synthesis [2,3]. The search was intentionally limited to infections not endemic in Europe; conditions with established local European transmission, including West Nile fever and tick-borne encephalitis, were therefore outside the scope of the targeted search string. Hepatitis A, while meeting the operational definition of an imported infection, was not systematically captured because its epidemiology is primarily documented through national notification registries rather than post-travel clinical cohort studies of the type eligible for this review.

This approach was adopted to preserve methodological coherence. Expanding the scope to all theoretically importable infections would have introduced substantial heterogeneity, reduced comparability across studies, and limited interpretability. The review therefore focused on the most consistently reported and clinically informative infections within the available pediatric evidence base.

### 2.5. Search Strategy

A systematic literature search was conducted in PubMed, Scopus, and the Cochrane Library. The search covered studies published between 2010 and 2025, with the final search executed on 26 December 2025.

To maximize sensitivity, database-specific search strategies were applied. In PubMed, Medical Subject Headings (MeSH) were combined with free-text terms in titles and abstracts. In Scopus, searches were performed using TITLE-ABS-KEY fields to account for the absence of a controlled vocabulary. In the Cochrane Library, exploded MeSH descriptors were combined with keyword-based searches.

Search strategies integrated terms related to imported infectious diseases, pediatric populations, travel, and major infection groups, including malaria, arboviral and other viral infections, such as dengue or chikungunya, enteric infections, as well as parasitic diseases.

Searches were limited to human studies published in English. To ensure completeness, backward citation searching of included studies and relevant reviews was performed. The full database-specific search strategies used for PubMed, Scopus, and the Cochrane Library are provided in Supplementary Table S1.

### 2.6. Study Selection

Study selection was performed independently by two reviewers (J.M.B. and J.J.) using the Rayyan web-based platform, which enabled blinded screening. The selection process consisted of two stages: initial screening of titles and abstracts, followed by full-text assessment of potentially eligible studies.

Eligibility criteria were predefined and applied consistently at both stages. Discrepancies between reviewers were resolved through discussion and consensus. When necessary, a third reviewer (J.N.) was consulted to ensure consistency and minimize selection bias.

### 2.7. Data Extraction

Data were extracted using a structured standardized form. Extracted variables included study characteristics, country and years of data collection, pediatric sample size, infection categories, pathogen-specific diagnoses, Plasmodium species where relevant, travel destinations or exposure regions, reasons for travel when available, and selected clinical outcomes such as hospitalization.

Because reporting formats varied substantially across studies, only data that could be clearly attributed to pediatric patients were used for quantitative aggregation. When studies included both adults and children, a two-step procedure was applied. First, the study was assessed for the availability of separately reported pediatric data. Second, if pediatric-specific results were clearly reported and numerically extractable, either in the main text, tables, or Supplementary Materials, only those data were extracted and incorporated into the descriptive synthesis. If disaggregated pediatric data were unavailable for a given variable, the study was excluded from quantitative synthesis for that specific variable, but retained in the review if pediatric data were available for other variables. Studies in which pediatric data could not be separated from adult data for any variable were excluded from the review entirely at the full-text screening stage.

### 2.8. Data Standardization

To facilitate comparison across heterogeneous studies, infections were harmonized into predefined analytical categories. Malaria was treated separately and, where possible, resolved to species level. Non-malarial infections were categorized into bacterial, parasitic, arboviral, and other etiological groups. Syndromic categories such as diarrhea or gastroenteritis were distinguished from microbiologically defined pathogens and were analyzed separately when necessary to avoid conflating clinical presentation with confirmed etiology.

Travel destinations were standardized into broad geographic categories comprising Africa, Asia, the Americas/Caribbean, the Middle East, Europe, Oceania/Pacific, and unknown or mixed exposure regions. The Americas/Caribbean category was used only as a broad descriptive category for high-level visualization and was not intended to imply epidemiological or clinical homogeneity across North America, Latin America, South America, or the Caribbean. Where the original studies provided more detailed subregional information, these data were preserved in Supplementary Table S6. Broad categories reported in the original studies were retained as reported and were not arbitrarily redistributed into narrower subregions. When individual countries were listed, they were assigned to these broader regions according to standard geographic classification.

### 2.9. Data Aggregation Strategy

Given the substantial heterogeneity in study design, reporting structure, and diagnostic detail, data aggregation followed a conservative, rule-based approach. Only pediatric-specific numerical data were included in pooled descriptive counts. Percentage-only data were excluded unless absolute values could be directly derived from reported denominators. Studies reporting mixed adult–pediatric populations without separate pediatric data were excluded from quantitative synthesis for the affected variables.

Data were extracted and analyzed across three analytical dimensions: pathogen distribution, travel characteristics (including reason for travel), and geographic exposure. Each dimension was constructed using only studies providing extractable numerical data for the given variable and was analyzed independently.

Pathogen-level aggregation was restricted to etiologically defined pathogens or clearly reported pathogen groups. Syndromic categories, such as acute diarrhea or gastroenteritis, were not merged with microbiologically confirmed diagnoses and were analyzed separately. Overlapping or partially overlapping classifications, like enteroinvasive *Escherichia coli* or *Shigella*, were retained as reported and were not further harmonized across studies.

For malaria-focused studies, species-level data were extracted as reported. Individual Plasmodium species were counted only when explicitly provided, while mixed and unspecified infections were retained as separate categories and not redistributed across species.

Travel-related variables, including reason for travel and destination, were aggregated using standardized categories where possible. Cases with incomplete, multiple, or unclear attribution were classified as unknown or mixed and were not reassigned. Regional aggregation was limited to studies reporting numerical pediatric travel data and was harmonized into broad geographic regions.

Because individual studies contributed data to different analytical dimensions and varied in scope and reporting completeness, the resulting pooled counts do not represent a single unified population. Accordingly, values presented across tables and figures are not strictly additive and should be interpreted as descriptive summaries of heterogeneous and partially overlapping datasets.

Detailed study-level extracted data and category harmonization rules are provided in Supplementary Table S6.

### 2.10. Risk-of-Bias Assessment

Risk of bias was assessed to characterize the methodological quality of the included evidence rather than to exclude studies solely on the basis of design type. Owing to the heterogeneity of study designs, four appraisal tools were applied: the Newcastle–Ottawa Scale (NOS) for cohort studies [12], an adapted version of the Newcastle–Ottawa Scale for cross-sectional studies [13], the JBI Critical Appraisal Checklist for Case Series [14], and the JBI Critical Appraisal Checklist for Studies Reporting Prevalence Data [15]. Assessment of the risk of bias was conducted independently by three reviewers (DG, JB, and JJ) for each outcome of all the selected studies. Any discrepancies were resolved by discussion until a consensus was reached. Detailed study-level quality assessments are presented in Supplementary Tables S2–S5, organized according to the appraisal tool used.

For NOS-assessed studies, a score of ≥5 out of nine stars was considered indicative of acceptable methodological quality. For JBI-assessed studies, no universal numerical threshold was applied given the checklist format. Instead, retention decisions were based on whether three key bias-relevant domains were met. Those comprised case ascertainment, the consecutive or complete inclusion of cases, and the clarity of case definition. Studies failing to meet these core criteria would have been flagged for exclusion or sensitivity analysis. In the present synthesis, no study was excluded on these grounds, as all retained studies demonstrated adequate case ascertainment and clearly reported case definitions. A post hoc sensitivity analysis restricted to studies meeting the above quality thresholds confirmed that the principal descriptive findings were not materially altered by the inclusion of lower-rated studies (see Section 4.7).

### 2.11. AI-Assisted Data Visualization and Verification

Data visualization was performed using Claude Sonnet 4.6 (Anthropic, San Francisco, CA, USA), a large language model-based tool, under direct and continuous supervision of the authorship team. The use of this tool was limited exclusively to the graphical rendering of numerical data that had been previously extracted, harmonized, and verified through the standard data extraction process described in Section 2.7, Section 2.8 and Section 2.9. No analytical, interpretive, or synthesis decisions were delegated to the AI tool at any stage.

Raw numerical data were manually transcribed from the study-level extracted dataset into structured natural-language prompts specifying chart type, axis labels, category names, and numerical values. No raw data files were uploaded directly to the model. Prompts were structured to minimize ambiguity. Each prompt specified the complete dataset, desired chart type, axis labels, color scheme, and annotation format explicitly. The model was not used to interpret, aggregate, or analyze data, and no AI-generated text appears in the manuscript.

Following figure generation, all visualizations were independently verified by two members of the authorship team (J.N. and J.M.B.) through direct, value-by-value comparison of all displayed numerical annotations against the corresponding source data in Table 1, Table 2 and Table 3 and Supplementary Table S6. Any discrepancies identified during verification were corrected prior to inclusion in the manuscript.

### 2.12. Data Synthesis

The synthesis of results was conducted using a structured descriptive approach tailored to the heterogeneity of the included studies. Data from the three analytical datasets (malaria-specific, non-malarial pathogen, and regional datasets) were analyzed separately to preserve conceptual clarity and avoid inappropriate merging of fundamentally different data types.

Aggregated counts were used to describe the frequency and distribution of infections and regions of acquisition. Emphasis was placed on identifying consistent epidemiological patterns across studies rather than deriving pooled effect estimates.

The synthesis prioritized comparability and transparency, with results interpreted in the context of differences in study design, reporting practices, and diagnostic approaches. Visual representations were used to support interpretation and to illustrate key patterns in the data.

Due to substantial heterogeneity in study populations, outcome definitions, and reporting formats, quantitative meta-analysis was not considered appropriate.

## 3. Results

### 3.1. Study Selection

**Figure 1:**
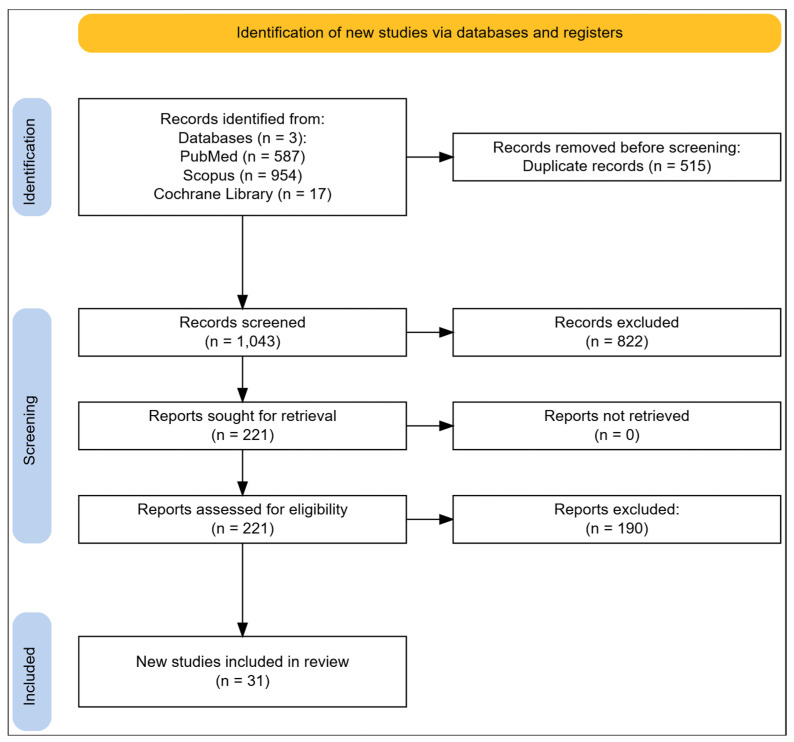
PRISMA flow diagram of the study selection process [16].

The initial search yielded 1558 records (587 from PubMed, 954 from Scopus and 17 from the Cochrane Library). Following the removal of duplicates, 1043 studies remained for screening. Based on abstract analysis, 822 articles were rejected for not satisfying the inclusion criteria. A rigorous full-text evaluation of the remaining 221 studies resulted in the exclusion of a further 190 reports, leaving a final total of 31 studies for inclusion in this systematic review. The full search process is pictured in Figure 1.

### 3.2. Study Characteristics

The included studies represented a heterogeneous but complementary evidence base on imported infectious diseases in children returning to Europe [11,17,18,19,20,21,22,23,24,25,26,27,28,29,30,31,32,33,34,35,36,37,38,39,40,41,42,43,44,45,46]. They were conducted across multiple European countries, most commonly Belgium, France, Spain, Italy, Germany, Greece, Sweden, and the United Kingdom/Ireland, and covered a broad temporal range from the early 1990s to 2024 [11,17,18,19,20,21,22,23,24,28,29,33]. Study settings included national surveillance systems, multicenter observational cohorts, hospital-based case series, and outpatient post-travel evaluations [23,24,25,26,28].

The overall number of pediatric cases varied markedly between studies, ranging from small single-center cohorts of fewer than 50 children to large national datasets including several thousand pediatric cases [21,23,27,28]. The evidence base comprised three broad study types: malaria-specific cohorts [17,18,19,20,21,23,28,29,30,31,32,33,34,46], mixed or syndromic cohorts assessing malaria together with other imported infections [11,22,24,25,26,35,36] and disease-specific non-malarial studies focusing on selected bacterial, parasitic, or arboviral infections [27,37,38,39,40,41,42,43,44,45].

Several studies included mixed adult–pediatric populations; only pediatric-specific data were extracted where separable and incorporated into the descriptive synthesis [23,30,31,37,40,44]. For studies in which pediatric cases represented a minority of the total cohort, such as Sondén et al. [22], where children aged 1–17 years constituted approximately 10% of a cohort of 2441 febrile returning travelers, pediatric-specific numerical data were extracted where explicitly reported and used only for variables for which separate pediatric counts were available. Variables reported only for the combined cohort were excluded from quantitative synthesis. Studies marked with an asterisk in Table 1, Table 2 and Table 3 indicate mixed adult–pediatric populations without fully separable pediatric analyses for all variables. In these cases, the reported pediatric proportion is indicated in the sample size column to allow readers to assess the degree of pediatric specificity. Overall, the included studies provide both high-volume epidemiological data for major imported infections, particularly malaria, and smaller but clinically informative datasets covering the broader spectrum of travel-associated disease in children.

**Table 1:** Epidemiology, travel characteristics, and *Plasmodium* species distribution in pediatric-imported malaria cases in Europe.

Ref.	Year of Publication	Years in Which Data Gathered	Country	Sample Size (Number of Children)	Reported Pediatric Proportion (%)	Pediatric Population			
						Age Range of Population	Travel Destination/Areas	Reason of Travel	Plasmodium Species
Selimaj Kontoni et al. [17]	2023	2009–2019	Belgium	160 (160)	100%	5–191 months	Sub-Saharan Africa—154 (96%) Asia—6 (4%)	VFRs—109 (68%) Visitors or newly installed migrants—49 (31%) Tourist—2	*P. falciparum*—144 (90%) *P. vivax*—10 (6%) *P. ovale*—3 (2%) *P. malariae*—1 mixed—2
Finale et al. [29]	2020	1989–2015	Italy	172 (172)	100%	0–14 years	North and West Africa—168 Asia—3 Not reported—1 Benin—3 Burkina Faso—16 Cameroon—10 Congo—8 Ivory Coast—27 Ghana—9 Equatorial Guinea—3 India—3 Kenya—2 Liberia—1 Madagascar—2 Mali—4 Nigeria—73 Not reported—1 Senegal—9 Togo—1	Immigration—26 (15%) Parental work—2 (1.2%) Residence—3 (1.7%) Return of origin country—102 (59.3%) Tourism—20 (11.6%) Other—2 (1.2%) Does not know—17 (9.9%)	*P. falciparum*—150 *P. ovale*—8 *P. vivax*—3 Mixed—3 Not specified—9
Luise et al. [30]	2017	2005–2015	Italy	172 (48)	27.9%	0–13 years	Africa—48 (100%)	VFRs—42 (87.4%) Tourist—3 (6.3%) Immigration—3 (6.3%)	*P. falciparum*—46 (95.8%) *P. ovale*—2 (4.2%)
Agagliati et al. [20]	2022	2007–2019	Italy	72 (72)	100%	8 months–14.7 years	Africa—71 (98.6%) India—1 (1.4%) Nigeria, Ghana, Ivory Coast, Senegal, Dem. Republic of Congo and Cameroon	VFRs—53 (73.6%) Immigration—10 (13.9%) Adoption—4 (5.55%) Visitors—2 (2.8%) Non known—3 (4.2%)	*P. falciparum*—68 (94.4%) *P. ovale*—2 (2.8%) *P. vivax*—1 (1.4%)- from India Not specified—1 (1.4%)
Zanotti et al. [31]	2017	1999–2016	Italy	1200 (225)	18.8%	0–16 years	Sub-Saharan Africa—185 (16.9%) Indian subcontinent—38 (16.9%) Unknown—2 (0.9%)	VFRs—173 (76.9%) Immigration—45 (20%) Unknown—6 (2.7%)	*P. falciparum*—170 (75.6%) *P. vivax*—45 (20%) *P. ovale*—9 (4%) *P. malariae*—1 (0.4%)
Mornand et al. [28]	2017	1996–2005	France	4150 (4150)	100%	0–15 years	Africa—4136 (99.6%) Ivory Coast (22%) Comoros Island (20%) Mali (16%) Senegal (13%) Cameroon (9%) Asia—8 Caribbean—3 Latin America—2 Pacific—1	No data available	*P. falciparum*—4150 (100%)
Sánchez Soto et al. [32]	2016	2007–2013	Spain	147 (147)	100%	<16 years	Mainly Sub-Saharan Africa (45% Equatorial Guinea) Africa—144 (98%) Asia—3 (2%)	VFRs—66 (45.8%)	*P. falciparum*—135 (90.6%) *P. ovale*—10 (6.7%) *P. malariae*—3 (2%) Mixed—9 (6%) Unknown—8 (5.4%)
Vygen–Bonnet Stark et al. [23]	2018	2001–2016	Germany	11,678 2001–2013 = 8492 2014–2016 = 3008	2001–2013 (9.7%) 2014–2016 (16.9%)	<18 years	Not specified for pediatric population. For whole group mainly Africa (88.8%)	No data available	Not specified for pediatric population
Maltezou et al. [21]	2013	1972–2012	Greece	21 (21)	100%	18 months–13 years	Pakistan—4 Nigeria—2 Ghana—2 India—2 Afghanistan—2 Congo—1 Tanzania—1 Uganda—1 Zambia—1	Immigration—7 VFRs—6 Repatriation to Greece—1 Occupation—1 Vacation—1	*P. vivax*—12 *P. falciparum*—7 Mixed—2
Garcia–Villarrubia et al. [19]	2011	1990–2008	Spain	174 (174 *)	100% *	<20 years *	Africa—146 (83.9%) Asia—15 (8.6%) America—3 (1.7%) Unknown—10 (5.7%)	Natives—31 Immigrants—143 VFRs—108 (62.1%)	*P. falciparum*—121 (69.5%) *P. vivax*—25 (14.4%) *P. ovale*—10 (5.7%) *P. malariae*—8 (4.6%) Mixed—5 (2.9%) Unknown—5 (2.9%)
Arnaez et al. [33]	2010	1995–2007	Spain	60 (60)	100%	17 days–14 years	Equatorial Guinea—55 (92%) Gabon—2 (3%) Nigeria—2 (3%) Ecuador—1 (2%) Natives Equatorial Guinea—14 (100%)	VFRs—14 Immigrants—46	*P. falciparum*—43 (72%) *P. ovale*—6 (10%) *P. vivax*—1 (2%) Mixed—5 (8%) Unknown—5 (8%) Natives *P. falciparum*—11 (79%) *P. ovale* 3 (21%)
Dubos et al. [18]	2010	2000–2006	France	133 (133)	100%	<18 years	Data available for 120 patients West Africa—86 (78%) East Africa—21 (19%) Asia—3 (3%) Unknown—10 (8%)	No data available	*P. falciparum*—83% *P. vivax*—7% *P. ovale*—3% Mixed—6%
Ladhani et al. [46]	2010	2006–2007	United Kingdom, Republic of Ireland	172 (172)	100%	<16 years	All cases—Africa—159 (92.4%) Travelers—Africa—105 (89.4%)	Travelers—117	Predominantly *P. falciparum*;
Minodier et al. [34]	2011	2004–2009	France	95 (95)	100%	3 months–16 years	Comoros—90% Madagascar—4% Togo—2% Ivory Coast—1% Senegal—1% Mali—1% French Guyana—1%	No data available	*P. falciparum*—100% + *P. ovale*—3 (3%) + *P. vivax*—1 (1%)

Abbreviations: VFR—visiting friends and relatives; %—percentage. Sample size is presented as total cohort with the number of children in parentheses. * Study included despite an upper age limit < 20 years, as the cohort predominantly represents pediatric and adolescent patients and results are consistent with pediatric epidemiological patterns. +In Minodier et al., three ***P. ovale*** and one ***P. vivax*** infections were reported as coinfections in patients with confirmed ***P. falciparum*** malaria [34].

**Table 2:** Spectrum, epidemiology, and travel characteristics of imported infectious diseases in children returning to Europe in mixed or post-travel clinical cohorts.

Ref.	Year of Publication	Years in Which Data Gathered	Country	Sample Size (Number of Children)	Reported Pediatric Proportion (%)	Pediatric Population			
						Age Range of Population	Infection Type/s	Travel Destination/Areas	Reason of Travel
Sondén et al. [22]	2025	2014–2024	Sweden	2441 (244) Fever in travelers returning from tropical and subtropical areas	10%	1–17 years	Malaria—14	Sub-Saharan Africa—13 North Africa and Middle East—1	
							Dengue—5	Sub-Saharan Africa—1 Asia and Pacific—4	
							Typhoid fever—3	Sub-Saharan Africa—1 Asia and Pacific—1 North Africa and Middle East—1 North America—1	
							Leptospirosis—1	Asia and Pacific—1	
							Gastroenteritis—37	Sub-Saharan Africa—15 Asia and Pacific—13 North Africa and Middle East—9	
Badillo Navarro et al. [35]	2024	March 2012–December 2019	Spain	100	100%	<16 years	Malaria—24 Dengue—2 Chikungunya—1 Amoebic liver abscess—1	Sub-Saharan Africa—87 Dominican Republic—8 Venezuela—2 Costa Rica—1 Morocco—1 Paraguay—1	VFR—43 Immigrant—45 Tourism—9 Not documented—3
Bird et al. [11]	2024	2016–2017	UK	1414	100%	<16 years	Malaria—60 Typhoid—21 Dengue—4 Leishmaniasis—1 Gastroenteritis—178 Dysentery—12	South Asia—44% Sub-Saharan Africa—33.3% Unknown—15% East and Southeast Asia, Oceania—4% South America—3%	Reason for travel not captured
Torres–Fernandez et al. [26]	2020	July 2002–July 2018	Spain	188	100%	<18 years	Malaria—39 (20.7%) Probably acute viral gastroenteritis—14 (7.4%) Bacterial gastroenteritis—5 (2.7%) Typhoid fever—3 (1.6%) Parasitic gastroenteritis—3 (1.6%)	Sub-Saharan Africa—103 (54.8%) South America—56 (29.8%) Asia—9 (4.8%) Non-available data—20 (10.6%)	VFR—89 (47.3%) Recently arrived migrants—61 (32.4%) VFR born abroad—17 (9%) Tourism—4 (2.1%)
Herbinger et al. [25]	2012	1999–2009	Germany	890	100% (0–19 years)	<20 years	For 774 travelers with German origin:		
							Giardiasis—62 (8%) Schistosomiasis—32 (4.1%) Superinfected insect bites—30 (3.9%) *Campylobacter* enteritis—29 (3.7%) *Salmonella* enteritis—27 (3.5%) *Cutaneous larva migrans*—24 (3.1%) Amebiasis—19 (2.5%) Dengue fever—18 (2.3%) Malaria—15 (1.9%) *Shigella* enteritis—11 (1.4%)	Africa—359 (46.4%) Asia—269 (34.8%) Latin America—146 (18.9%)	ATB—253 (32.7%) VFR—228 (29.5%) Package tour—147 (19.0%) Business trip—50 (6.5%) Immigration—32 (4.1%) Missionary/volunteer—23 (3.0%) Exchange program—17 (2.2%)
Naudin et al. [24]	2012	July–December 2007	France	538	100%	<18 years	acute diarrhea (27.1%) malaria (9.5%)	North Africa—214 (39.8%) Sub-Saharan Africa—185 (34.4%) Middle East—24 (4.5%) West Indies—18 (3.3%) Central Asia—16 (3%) South East Asia—6 (1.1%) Central and South America—4 (0.8%) North America—2 (0.4%) Russia—1 (0.2%) Oceania—1 (0.2%)	Traveler—474 (88.1%) Migrant—24 (4.5%) Visitor—40 (7.4%)
Satarvandi et al. [36]	2024	2015–2020	Sweden	2315 (202)	9%	1–17 years	Malaria—12 (5.9%) Dengue—4 (1.9%) Gastroenteritis clinical diagnosis—26 (12.9%) Gastroenteritis confirmed etiology—8 (3.9%) Typhoid fever—3 (1.5%)	Sub-Saharan Africa—90 (44.6%) Asia and Pacific—66 (32.7%) South/Latin America—10 (5.0%) Middle East/North Africa—27 (13.4%) North America (subtropical area)—1 (0.5%)	Reason for travel not captured

Abbreviations: VFR—visiting friends and relatives; ATB—adventure travel/backpacking; %—percentage. Sample size is presented as total cohort with the number of children in parentheses where applicable. Incidence (%) refers to the proportion of pediatric cases or infection-specific frequency, as reported in the original studies. Infection categories, travel regions, and reasons for travel are reported according to the original study definitions.

**Table 3:** Spectrum, epidemiology, and travel characteristics of non-malarial imported infectious diseases in children returning to Europe.

Ref.	Year of Publication	Years in Which Data Gathered	Country	Sample Size (Number of Children)	Reported Pediatric Proportion (%)	Pediatric Population			
						Age Range of Population	Infection Type/s	Travel Destination/Areas	Reason of Travel
Enkelmann et al. [37]	2025	2001–2023	Germany	2251 (618) * only imported cases	27.5%	<18 years	*S.* Paratyphi A—69	Asia—81.4% Africa—9.7% Americas—6.4% Europe—1.9% Exposure in multiple continents—0.7% (data for children and adults) *	Not specified
							*S.* Paratyphi B—180	Asia—97.2% Africa—1.6% America—0.4% Europe—0.6% Exposure in multiple continents—0.2% (data for children and adults) *	
							*S.* Typhi—356	Asia—74.6% Africa—1.8% America—13.1% Europe—9.9% Exposure in multiple continents—0.6% (data for children and adults) *	
Ria et al. [39]	2024	2023	Italy	43 (43)	100%	7 months–17.3 years	Shiga toxin-producing *E. coli* (STEC)	Egypt—12 None travel abroad —23 European country—8	Not specified
Pouletty et al. [38]	2018	1 August 2014–15 October 2015	France	59 (59)	100%	<18 years	Bacteria EAggEC—32 (54%) EPEC—26 (44%) ETEC—19 (32%) EIEC/*Shigella*—16 (27%) STEC—4 (6%) *Salmonella* spp.—16 (27%) *Campylobacter jejuni*—10 (17%) *Clostridium difficile*—3 (5%) Virus Sapovirus—11 (18%) Norovirus—10 (17%) Rotavirus—9 (15%) Astrovirus—4 (6%) Adenovirus—2 (3%) Parasites Cryptosporidium—11 (18%) Giardia—8 (13%)	Sub-Saharan Africa—29 (49%) North Africa—25 (42%) Asia—4 (6%) South Africa—1 (1.7%)	VFRs—35 (59%) Tourism—24 (40%)
Söbirk et al. [40]	2018	1993–2016	Sweden	182 (58)	31.9%	<18 years	Leishmaniasis *L. tropica*—77 *L. (Viannia)* subgenus—34 *L. major*—31 *L. donovani* complex—22 *L. aethiopica*—3 *L. mexicana*—2 Not typed—13 (data for children and adults) *	Asia—102 South America—27 Africa—13 Europe—13 Central America—10 Unknown/missing—17 (data for children and adults) *	Not specified
Pommelet et al. [41]	2018	1993–2015	France	50 (50)	100%	<18 years	*S.* Typhi—44 (88%) *S.* Paratyphi—6 (12%)	Sub-saharan Africa—14 (34%) Indian Subcontinent—14 (34%) North Africa—11 (27%) Middle East—1 (2%) Other—1 (2%) Domestically acquired infection—7 (14%)	VFR—29 (85%) Tourism—2 (6%) Other—3 (9%)
Soriano–Arandes et al. [42]	2016	2009–2013	Spain	606 (606)	100%	<16 years	Protozoan—142 (23.4%) Helminthes—97 (16%) Bacterial—13 (2.2%) Unknown—354 (58.4%) Giardiasis—61 (10.1%)	Europe and North America—10 (1.65%) Sub-Saharan Africa—184 (30.36%) North Africa—22 (3.63%) Latin America and Caribbean—220 (36.3%) Indian Subcontinent and Asia—170 (28.05%)	Tourism—34 (5.6%) VFR traveler—131 (21.6%) VFR immigrant—42 (6.9%) Immigrant—399 (65.9%)
Mellado–Sola et al. [27]	2025	2015–2024	Spain	46 (46)	100%	<16 years	Dengue	Dominican Republic—10 (22%) Cuba—7 (15%) Paraguay—4 (8.6%) Colombia—4 (8.6%) Central and South American countries, Southeast and Central Asia—7 (15%) Equatorial Guinea—1 (2%)	VFRs—26 (56%) Immigrants—11 (24%) Tourists—7 (15%) Unknown—2 (5%)
Mendoza–Palomar et al. [43]	2020	2010–2017	Spain	51 (51)	100%	<18 years	*S. haematobium*—43% *S. mansoni*—24% *S. intercalatum*—24%	Africa—100%	Immigrants—43 VFR—4 Tourist—4
Guery et al. [44]	2021	2006–2019	10 centers in 7 European countries	459 (106)	23%	<18 years	Leishmaniasis (Localized Cutaneous type—100%)	Syria—22 Tunisia—16 Morocco—15 Algeria—12 Senegal—12 Israel—6 Spain—5 Mauritania—5 Costa Rica—4 Pakistan—2 France—1 Peru—1 Egypt—1 Turkey—1 Iran—1 Mali—1 Ecuador—1	VFRs—60 Tourist—15 Migrant—25 N/A—3 Expatriate (worker, missionary)—1 Other—2
Cnops et al. [45]	2020	2017 (single exposure event)	Belgium	34 (18)	53%	5–15 years	Schistosomiasis	South Africa—18 (100%)	Tourist—100%

Abbreviations: VFR—visiting friends and relatives; N/A—not available; %—percentage; STEC—Shiga toxin-producing *Escherichia coli*; EAggEC—enteroaggregative *Escherichia coli*; EPEC—enteropathogenic *Escherichia coli*; ETEC—enterotoxigenic *Escherichia coli*; EIEC—enteroinvasive *Escherichia coli*. Data are reported according to the original study definitions. Where applicable, pediatric data were extracted from mixed adult–pediatric populations when separately available. Values are presented as absolute numbers and/or proportions as reported in the original studies. * Studies including mixed adult–pediatric populations without fully separable pediatric analyses.

**Figure 2:**
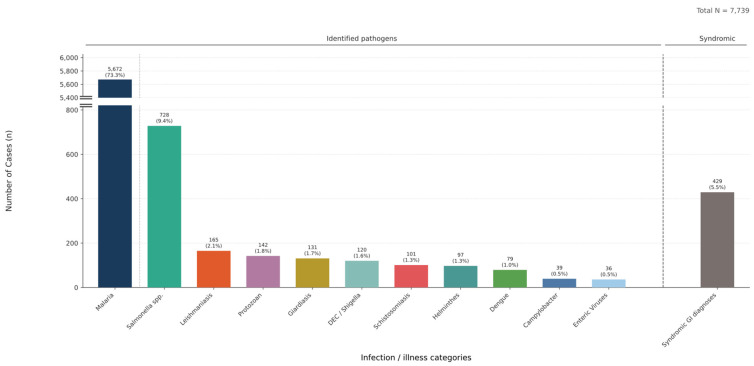
Distribution of travel-related infectious pathogens and illness categories reported in pediatric patients returning to Europe. Values represent aggregated descriptive counts extracted from eligible studies and include only pediatric cohorts or mixed-population studies in which pediatric data could be separately identified. “Identified pathogens” include microbiologically confirmed or specifically reported etiological diagnoses, whereas “Syndromic GI diagnoses” include clinically reported gastrointestinal syndromes without pathogen-specific confirmation. *Salmonella* spp. includes reported *Salmonella* Typhi, *Salmonella* Paratyphi (A/B or unspecified), *Salmonella enteritis*, and *Salmonella* spp. categories as classified in the original studies. DEC/*Shigella*—diarrheagenic *Escherichia coli*/*Shigella* group includes enteroaggregative *E. coli* (EAggEC), enteropathogenic *E. coli* (EPEC), enterotoxigenic *E. coli* (ETEC), enteroinvasive *E. coli* (EIEC), Shiga toxin-producing *E. coli* (STEC), and overlapping EIEC/*Shigella* categories as reported in the original studies. Helminths denote a grouped category of helminthic infections reported without species-level classification in one cohort. Schistosomiasis denotes separately reported confirmed schistosomal infections from dedicated cohorts. Because these two categories derive from different studies and reporting structures, they are shown separately for descriptive purposes and are not necessarily mutually exclusive. Enteric viruses include norovirus, rotavirus, sapovirus, astrovirus, and adenovirus where specified. Syndromic GI diagnoses include acute diarrhea, gastroenteritis, and dysentery categories reported without fully resolved pathogen attribution. Detailed category composition and study-level source data are provided in Supplementary Table S6. Percentages are based on the aggregated pathogen dataset and do not represent a single unified patient cohort. The apparent predominance of malaria (*n* = 5672; 73.3%) reflects two compounding factors of the disproportionate contribution of large national malaria surveillance cohorts to the aggregated dataset and a spectrum effect arising from the predominance of hospital-based study settings, which systematically overrepresent conditions requiring inpatient management relative to self-limiting conditions managed in primary care. This figure should therefore be interpreted as an illustration of the breadth of the reported pathogen spectrum across the available evidence base, rather than as a proportional representation of the relative clinical burden of individual conditions in unselected post-travel pediatric populations. Owing to substantial differences in diagnostic certainty across categories (malaria confirmed by standardized parasitological methods versus gastrointestinal conditions frequently reported on syndromic or heterogeneous diagnostic grounds), individual pathogen categories do not carry equal evidentiary weight and should not be directly compared as if diagnostic confirmation were uniform.

**Figure 3:**
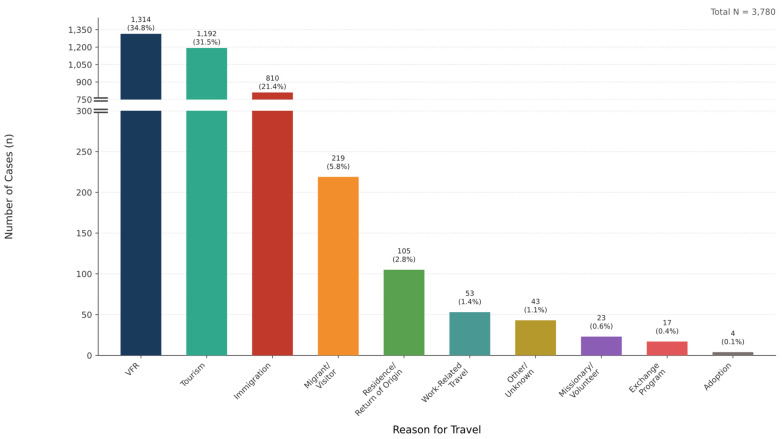
Distribution of reported travel reasons among pediatric patients with imported infectious diseases returning to Europe. Values represent aggregated descriptive counts extracted from eligible studies according to the original study definitions. Categories were harmonized where possible; however, terminology varied across studies and may include partially overlapping migration-related classifications. VFR—visiting friends and relatives. Detailed study-level extracted travel-purpose data and category harmonization are presented in Supplementary Table S6. Percentages are based on the aggregated reported counts and should be interpreted descriptively rather than as population incidence estimates.

**Figure 4:**
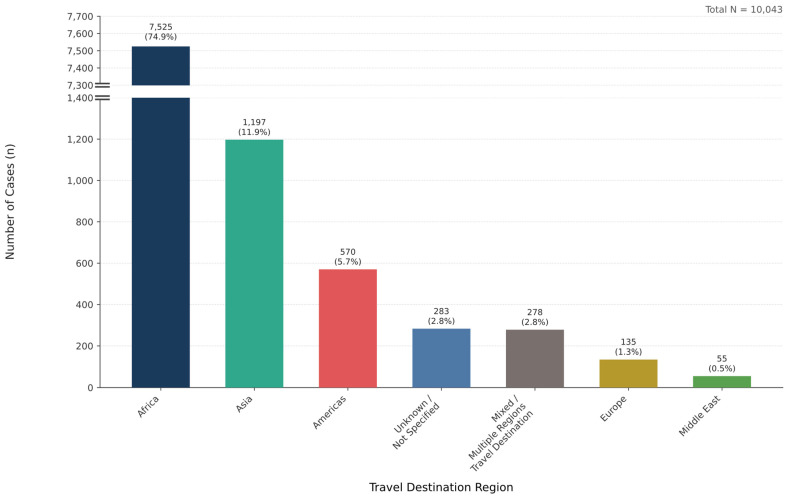
Geographic distribution of travel destinations associated with imported infectious diseases in pediatric patients returning to Europe. Bars represent aggregated reported travel exposures extracted from eligible studies. Africa (predominantly sub-Saharan Africa) accounted for the largest proportion of reported travel exposures in the included clinical cohorts, followed by Asia and the Americas. Smaller contributions were observed from Europe and the Middle East. This geographic distribution reflects both the distribution of VFR travel in the study populations and the predominance of malaria-specific cohorts in the dataset, rather than necessarily representing per-traveler infection risk. The Americas/Caribbean category is retained as an aggregate category in the figure for consistency with the high-level geographic visualization. It should not be interpreted as a clinically homogeneous risk region, because it combines epidemiologically distinct settings, including North America, Latin America, South America, and the Caribbean. More detailed subregional information available from the original studies is preserved in Supplementary Table S6. Broad categories reported in the original studies were retained as reported and were not arbitrarily redistributed into narrower subregions. The category “Europe” reflects 135 of 10,043 reported travel exposures (1.3%) classified under the Europe destination category in Supplementary Table S6. These exposures were contributed by six studies: Sondén et al. [22] (*n* = 7), Bird et al. [11] (approximately *n* = 14), Naudin et al. [24] (*n* = 67), Satarvandi et al. [36] (*n* = 8), Ria et al. [39] (*n* = 33), and Guery et al. [44] (*n* = 6). Soriano–Arandes et al. [42] additionally reported 10 exposures in a combined “Europe and North America” category; these were not included in the Europe-only count because they could not be reliably attributed to intra-European travel. Sensitivity checks excluding Europe-classified exposures, and a stricter check excluding both Europe-classified exposures and the combined Europe/North America category, did not alter the principal geographic pattern, with Africa and Asia remaining the dominant reported exposure regions. Unknown/Not specified—includes cases in which no destination was reported or insufficient geographic detail was provided in the original study. Mixed/Multiple regions—includes travelers with more than one reported destination, multi-country itineraries, or exposure categories spanning several regions. Detailed study-level extracted destination data are presented in Supplementary Table S6. Percentages are based on the aggregated reported travel-destination dataset (Total *n* = 10,043) and do not represent a single unified patient cohort.

Across the full aggregated dataset, malaria was the most frequently reported infection, accounting for the majority of pathogen-specific cases (*n* = 5672; 73.3% of the aggregated pathogen dataset), followed by *Salmonella* spp. (*n* = 728; 9.4%), Syndromic GI diagnoses (*n* = 429; 5.5%), Gastroenteritis (*n* = 272; 3.5%), and Leishmaniasis (*n* = 165; 2.1%), with the remaining pathogen categories each accounting for less than 2% of the total (Figure 2). With regard to travel purpose, VFR travel was the dominant reported category across studies where this information was available, representing the largest proportion of reported cases in both malaria-specific and non-malarial cohorts (Figure 3). Geographically, Africa, predominantly sub-Saharan Africa, accounted for the largest share of reported travel destinations (74.9%), followed by Asia (9.4%) and the Americas (5.7%) (Figure 4). These aggregate patterns are discussed in detail in the following sections, stratified by infection category, travel destination, and travel purpose. As noted in Section 2.12, these figures represent aggregated descriptive counts across heterogeneous study types and should not be interpreted as proportional representations of clinical burden in unselected pediatric post-travel populations.

### 3.3. Imported Malaria

Imported malaria was the most consistently investigated infection across the included studies and the condition for which the most clinically comparable evidence was available [17,19,28,31,32]. Most malaria studies were retrospective hospital-based cohorts, multicenter observational series, or national surveillance datasets, together covering a wide time span and a large cumulative number of pediatric cases [23,28,31]. In mixed-age cohorts, children accounted for a substantial proportion of cases, including 18.8% in the Brescia series and 27.9% in the Padua cohort, while German surveillance data showed an increase in the pediatric proportion over time, from 9.7% in 2001–2013 to 16.9% in 2014–2016 [23,30,31]. In contrast, several studies were restricted exclusively to pediatric cases and confirmed that children constitute a clinically relevant subgroup of imported malaria in Europe [17,18,20,21,28,29,32,33,34,46].

A highly consistent geographic pattern emerged across studies, with sub-Saharan Africa representing the most frequently reported region of exposure in clinical cohorts [17,19,20,28,32,46]. In most cohorts, African exposure accounted for the clear majority of pediatric malaria cases, typically ranging from approximately 84% to nearly 100% [20,28,32,46]. Frequently reported source countries included Equatorial Guinea, Nigeria, Ghana, Côte d’Ivoire, Senegal, Cameroon, Mali, Comoros, and the Democratic Republic of Congo [19,21,28,29,33]. Travel to Asia or Latin America was much less frequently reported and was more commonly recorded in association with non-falciparum malaria [19,21,31]. The reason for travel was an important correlate of case distribution in the included studies. Where reported, visiting friends and relatives (VFR) was the dominant travel category, accounting for approximately 45.8% to 87.4% of cases across disaggregated cohorts [17,19,20,30,32]. Even in studies using broader or alternative categories, such as return to country of origin, immigration, or residence abroad, the same overall pattern was evident, indicating that most pediatric malaria cases occurred in children from migrant families traveling to endemic settings rather than in conventional tourists [21,29,33]. Although refugee populations were not part of the predefined eligibility criteria, some studies used grouped migration-related categories that may have partly overlapped with broader mobility patterns.

With regard to species distribution, *Plasmodium falciparum* overwhelmingly predominated across studies, accounting for most pediatric-imported malaria cases in Europe [17,20,28,32,34]. Non-falciparum infections were less frequent and mainly included *P. vivax* and *P. ovale*, with occasional *P. malariae* and mixed-species infections [17,19,21,31,33]. A relatively higher proportion of *P. vivax* was observed in cohorts including travel to Asia or other non-African regions [19,21,31], consistent with the geographic distribution of malaria species globally.

Compared with the broader imported-infection literature, malaria studies showed greater diagnostic uniformity, relying mainly on microscopy, often supplemented by rapid diagnostic testing and, in selected cohorts, molecular methods. This relative methodological consistency supports the cross-study comparison of species distribution and broad epidemiological patterns. However, the reporting of prophylaxis use, timing of diagnosis, and severity markers was less standardized, limiting more detailed cross-study comparisons across datasets. Overall, the available evidence indicates that pediatric-imported malaria in Europe follows a stable epidemiological pattern defined by African acquisition, predominance of VFR travel, and *P. falciparum* as the leading infecting species. These patterns, however, apply specifically to malaria-focused cohorts and should not be generalized to the overall spectrum of post-travel illness in unselected pediatric populations, where gastrointestinal and other non-malarial conditions predominate [11,24,25].

### 3.4. Imported Infections in Mixed or Syndromic Cohorts

Studies in this subgroup predominantly included children presenting after travel with febrile illness or broader post-travel clinical syndromes, although some cohorts also included diagnosis-based or mixed clinical populations (Table 2). Across cohorts from Sweden, Spain, the United Kingdom, France, and Germany, malaria remained an important diagnosis but did not account for the majority of cases in most mixed or syndromic cohorts [11,22,24,25,26,35,36]. Instead, these studies demonstrated that non-malarial imported infections, particularly gastrointestinal illnesses, represented a major share of post-travel morbidity in children.

Malaria accounted for a variable but generally limited proportion of cases in these broader cohorts, ranging from 1.9% in the German outpatient post-travel cohort to 20.7% in the Spanish febrile cohort, with intermediate proportions reported in French and Swedish datasets [24,25,26,36]. In contrast, gastrointestinal conditions were frequently among the leading diagnoses. Acute diarrhea accounted for 27.1% of cases in the French cohort [24], while gastroenteritis was the most common diagnosis in the UK study [11]. In the Swedish and Spanish mixed cohorts, gastroenteritis, dengue, typhoid fever, and other systemic imported infections were recurrent but less frequent than the overall burden of gastrointestinal illness [22,26,35,36].

The geographic pattern in these studies was broader than in malaria-specific cohorts but still dominated by sub-Saharan Africa and Asia, with additional contributions from Latin America, the Middle East/North Africa, and, less commonly, other regions [11,22,24,25,26,35,36]. Where reported, VFR travel and migration-related categories were common, again indicating that imported infections disproportionately affected children with family or migration links to endemic areas [25,26,35]. However, travel purpose was not consistently captured across all studies [11,22,36].

Taken together, these data show that while malaria remains a key diagnosis requiring systematic exclusion in returning children, it represents only one component of a much broader post-travel infectious spectrum. In unselected or syndromic cohorts, non-malarial conditions—especially gastrointestinal and febrile illnesses—predominate, supporting a broad clinical and microbiological diagnostic approach rather than a malaria-centered framework alone.

### 3.5. Disease-Specific Non-Malarial Imported Infections

The studies in this subgroup focused on specific imported infections other than malaria and were generally more pathogen-oriented than the mixed febrile-traveler cohorts [27,37,38,39,40,41,42,43,44,45]. Compared with the malaria literature, these datasets were usually smaller and more heterogeneous in both design and case selection, but they provided valuable detail on distinct clinical entities, including enteric fever, diarrheal infections, leishmaniasis, schistosomiasis, dengue, and protozoal or helminthic infections.

Enteric and gastrointestinal infections were particularly prominent in this group. Pediatric enteric fever due to *Salmonella enterica* serovars Typhi and Paratyphi showed a strong geographic association with Asia, especially in the German surveillance dataset, where imported cases were overwhelmingly linked to Asian exposure [37]. Similarly, the French pediatric enteric fever cohort showed substantial contributions from both sub-Saharan Africa and the Indian subcontinent, with a marked predominance of VFR travel among those with available travel-purpose data [41]. Broader diarrheal cohorts also documented a wide spectrum of bacterial, viral, and parasitic pathogens, including enteroaggregative and enteropathogenic *Escherichia coli*, *Salmonella*, *Campylobacter*, Giardia, Cryptosporidium, norovirus, and rotavirus [38]. In the Spanish epidemiological cohort, protozoal and helminthic infections were also common, with substantial representation of Latin America/Caribbean, sub-Saharan Africa, and Asia [42].

Other disease-specific studies highlighted clear destination-linked patterns. Schistosomiasis was almost exclusively associated with African exposure [43], while a cluster related to a single exposure event in South Africa illustrated the importance of localized travel-associated outbreaks [45]. Leishmaniasis was linked mainly to Asia, North Africa, the Middle East, Africa, and selected Latin American settings, depending on species and cohort structure [40,44]. Dengue was primarily associated with Latin America and the Caribbean, although additional cases related to Asia and, less commonly, Africa were also reported [27]. Some datasets, such as the Italian STEC cohort, were not strictly travel-based and included a substantial proportion of domestically acquired or non-travel-associated cases, underscoring the need to distinguish imported disease from broader pediatric infectious presentations [39].

Where travel purpose was reported, VFR and migrant populations were again strongly represented [27,38,41,42,43,44]. This pattern was especially evident in studies of enteric fever, dengue, and parasitic infections, suggesting that reported case distribution is shaped not only by destination but also by travel context, duration of stay, and background links to endemic regions. Overall, these disease-specific studies show that non-malarial imported infections in children returning to Europe are highly diverse, encompassing invasive bacterial disease, diarrheal syndromes, parasitic infections, vector-borne disease, and travel-associated dermatological or hepatointestinal conditions.

### 3.6. Travel Characteristics: Reason for Travel

The distribution of travel-related infections according to reason for travel is presented in Figure 3. Across the included studies, visiting friends and relatives (VFR) constituted the most frequently reported travel category, followed by tourism- and migration-related travel. Other travel purposes, including business travel, volunteering, and adoption, were reported only sporadically.

This distribution indicates that reported cases were not evenly distributed across travel types and appear to be concentrated among children with closer or prolonged exposure to endemic environments. In contrast, conventional tourism was associated with a smaller proportion of reported cases, although differences in reporting practices between studies may have influenced these proportions.

However, these patterns should be interpreted with caution. Furthermore, the VFR category as reported across studies is internally heterogeneous, encompassing subpopulations that differ substantially in baseline immunity, exposure duration, and healthcare-seeking behavior. This heterogeneity limits the direct comparability of VFR proportions across studies and precludes drawing conclusions about any single VFR subpopulation. Although studies focusing exclusively on migrant populations were not eligible for inclusion, some cohorts used broader or overlapping classifications, such as VFR, immigration, or return-to-country-of-origin, which may have partially captured migration-related travel patterns. In addition, the definition and reporting of travel purpose were not standardized across studies, and a proportion of cases was categorized as unknown or not specified.

### 3.7. Cross-Study Methodological Heterogeneity

Substantial heterogeneity was observed across studies in terms of design, diagnostic methods, patient selection, and reporting. Most studies were retrospective and hospital-based, potentially overrepresenting more severe or clinically recognized infections, while outpatient and surveillance datasets captured different case mixes and were not directly comparable [23,25,28].

Diagnostic approaches varied substantially by pathogen group, creating a fundamental asymmetry in the evidentiary weight of individual categories. Malaria was almost universally confirmed using standardized parasitological methods, primarily blood film microscopy, often supplemented by rapid diagnostic testing and, in selected cohorts, PCR, providing a high and consistent level of diagnostic certainty across studies [17,20,28,32]. Enteric pathogens in the French diarrhoeal cohort were identified using a validated multiplex PCR panel applied to stool samples [38]. Enteric fever was confirmed by blood culture, occasionally supplemented by serology [37,41]. Dengue was diagnosed primarily through NS1 antigen detection and/or IgM/IgG serology, with PCR used in selected cases [27,36]. Serological methods cannot distinguish active from recent past infection without paired samples. Leishmaniasis was diagnosed using serology, PCR, or tissue microscopy depending on clinical form and cohort [40,44]. Schistosomiasis was confirmed by serology and/or direct parasitological methods [43,45]. Gastrointestinal conditions in syndromic cohorts were frequently reported on the basis of clinical presentation alone, without microbiological confirmation [11,24,25,26]. This diagnostic asymmetry means that confirmed pathogen counts for non-malarial conditions are not directly comparable to malaria counts and that the reported frequency of syndromic gastrointestinal illness is likely an underestimate given its dependence on the extent of microbiological investigation performed.

A narrative assessment of temporal trends across the included studies suggests that core epidemiological patterns, namely *P. falciparum* predominance and VFR overrepresentation, have remained broadly stable across the full temporal range of the evidence base. Notably, an emerging contribution of dengue and other arboviral infections is apparent in more recent studies, consistent with the geographic expansion of Aedes aegypti and improved molecular diagnostic capacity in European clinical settings. Formal quantitative trend analysis would require standardized denominators and temporally harmonized case definitions, and remains a priority for future dedicated research.

Age-stratified analysis by developmental subgroup, covering infants, school-aged children, and adolescents, was not feasible, as none of the 31 included studies provided systematically extractable age-disaggregated data across these categories. This represents a significant gap that limits the ability to characterize age-specific epidemiological patterns within the 0–18 year age range.

Additional heterogeneity resulted from the inconsistent classification of travel destinations, incomplete reporting of travel purpose, and inclusion of mixed adult–pediatric populations without fully separable pediatric data [23,37,40,44]. Findings should therefore be interpreted as a descriptive synthesis rather than a pooled quantitative estimate.

### 3.8. Geographic Distribution of Exposure/Travel Destinations

The distribution of travel destinations among pediatric patients was dominated by returns from sub-Saharan Africa, with Asia as the second-most frequent region and smaller contributions from the Americas, Europe, and the Middle East (Figure 4). The aggregated Americas/Caribbean category should be interpreted cautiously, as it combines exposures from epidemiologically distinct settings, including North America, Latin America, South America, and the Caribbean; more detailed subregional extraction, where available, is provided in Supplementary Table S6. This pattern aligns with the geographic structure of exposure observed across the included studies and underscores the role of destination-specific epidemiology in shaping the reported distribution of imported infections in clinical cohorts. These proportions reflect the composition of the included clinical cohorts rather than per-traveler incidence rates. In the absence of travel volume denominators, regional distributions cannot be interpreted as estimates of relative infection risk.

These findings highlight that travel destination is not only a descriptive variable but a clinically relevant factor that may guide early diagnostic reasoning, particularly in the context of region-specific pathogen prevalence.

### 3.9. Risk of Bias

After a thorough review, and taking into account the heterogeneity of the included studies, no studies were excluded solely on the basis of risk-of-bias assessment. Of the 31 selected studies, four were assessed using the Newcastle–Ottawa Scale for cross-sectional studies, with three rated as “Satisfactory studies” and one as “Good studies”. In addition, seven studies were evaluated using the Newcastle–Ottawa Scale for cohort studies, of which six were classified as “Good quality” and one as “Poor quality”. The Joanna Briggs Institute (JBI) Critical Appraisal Checklist for Case Series was used to assess 16 studies, while the remaining four were appraised using the JBI Critical Appraisal Checklist for Studies Reporting Prevalence Data. In both JBI tools, methodological quality was assessed by answering a series of questions with yes, no, unclear, or not applicable, followed by counting the number of “yes” responses. The checklist results were interpreted qualitatively, taking into account both the number of “yes” responses and the relevance of individual domains to the risk of bias. The decision to retain a study was based on an assessment of three key bias-relevant domains applicable across all design types: (1) clarity and consistency of case definition, (2) adequacy of case ascertainment, defined as whether consecutive or complete inclusion was applied or clearly justified, and (3) transparency of outcome reporting. All 31 retained studies demonstrated acceptable performance on these core domains. The one study rated as “Poor quality” on the NOS cohort scale [30] was retained because its methodological limitations were confined to comparability domains rather than to case definition or ascertainment, and because exclusion would not have materially affected the principal findings, given its limited contribution to the aggregated dataset. Overall, most studies received a predominance of “yes” responses, indicating moderate-to-high reporting transparency and acceptable methodological quality. Although not all studies achieved a high-quality rating, they were retained given the epidemiological nature of this review, as the data reported in these studies remained suitable for extraction and analysis despite certain methodological limitations.

Detailed study-level assessments are presented in Supplementary Table S2 for cross-sectional studies assessed with the Newcastle–Ottawa Scale, Supplementary Table S3 for cohort studies assessed with the Newcastle–Ottawa Scale, Supplementary Table S4 for studies assessed with the JBI Critical Appraisal Checklist for Case Series, and Supplementary Table S5 for studies assessed with the JBI Critical Appraisal Checklist for Studies Reporting Prevalence Data.

## 4. Discussion

### 4.1. Principal Findings

This systematic review provides a comprehensive synthesis of the epidemiology and spectrum of imported infectious diseases in pediatric populations returning to Europe. Across the included studies, malaria emerged as the dominant reported infection, accounting for the majority of pathogen-specific cases, followed by bacterial enteric infections, particularly *Salmonella* spp., and a range of parasitic diseases, including protozoan and helminthic infections [17,20,25,28,32,37,41,42]. Viral infections such as dengue were reported less frequently but remained clinically relevant in specific travel contexts [27,36].

Beyond pathogen-specific findings, the overall distribution of reported infectious entities reflects substantial heterogeneity in diagnostic approaches and reporting practices, with both etiologically confirmed infections and syndrome-based categories, such as gastroenteritis or acute diarrhea, contributing to the observed patterns. Consequently, the available evidence is better interpreted as reflecting reporting structures and clinical practice rather than strictly comparable case frequencies across pathogen groups.

From an epidemiological perspective, infections were most frequently observed in children returning from tropical and subtropical regions, with sub-Saharan Africa representing the predominant source of exposure across studies [17,23,25,28,46]. This pattern was closely linked to travel characteristics, as visiting friends and relatives (VFR) and immigration-related travel constituted the most common reasons for exposure, substantially exceeding tourism-related travel [17,20,26,32].

Taken together, these findings highlight a consistent epidemiological pattern characterized by the predominance of malaria, a substantial burden of enteric and parasitic infections, and a strong association with VFR-related travel to high-endemic regions, while also underscoring the impact of methodological heterogeneity on the interpretation of aggregated data.

It should be noted, however, that the apparent predominance of malaria in the aggregated pathogen dataset (Figure 2) is heavily influenced by the disproportionate weight of large national malaria surveillance cohorts. In broader post-travel cohorts capturing the full spectrum of illness in unselected pediatric populations, gastrointestinal conditions consistently represent the leading diagnostic category, and malaria accounts for a substantially smaller proportion of diagnoses [11,24,25]. An additional contributor to the apparent prominence of malaria in the aggregated dataset is a spectrum effect arising from the predominance of hospital-based study settings. Malaria and other severe infections requiring inpatient management will be systematically overrepresented in hospital cohorts relative to self-limiting conditions that resolve in the community or are managed in primary care. This ascertainment structure cannot be corrected at the level of secondary synthesis and should be borne in mind when interpreting all pathogen-level frequency data presented in this review.

### 4.2. Epidemiological Interpretation

The findings of this review highlight several important epidemiological patterns in imported infectious diseases among children and adolescents returning to Europe after international travel. Visiting-friends-and-relatives (VFR) travel was consistently overrepresented among reported cases [6,47,48]. Children traveling to their or their parents’ countries of origin are often exposed to higher endemic disease burdens, frequently remain in local community settings rather than tourist accommodations, and tend to have longer travel durations. Moreover, VFR travelers are less likely to seek pre-travel medical advice and preventive interventions, which are patterns that are consistently described in the literature as correlates of overrepresentation among reported cases of vaccine-preventable and vector-borne diseases [6,47,48].

It should be noted, however, that the VFR category as reported in the included studies is not internally uniform. It encompasses second-generation migrants visiting family abroad, first-generation migrants returning periodically and newly arrived migrants, which are subgroups that differ substantially in baseline immunity and prior exposure. The reported overrepresentation of VFR travel should therefore be interpreted as a broad epidemiological signal rather than a uniform characterization of a defined population.

The pathogen spectrum identified in this review should be contextualized against ECDC notification surveillance data for travel-associated vaccine-preventable diseases (VPDs), which are systematically absent from post-travel clinical cohort studies. Hepatitis A and measles are clinically relevant examples of travel-associated conditions that are underrepresented in this review because their epidemiology is primarily captured through national notification registries rather than post-travel clinical cohort studies of the type eligible for inclusion, despite both meeting the operational definition of an imported infection adopted in this review. For hepatitis A, between 2009 and 2015, 27.8% of confirmed EU/EEA cases were travel-associated, with VFR travelers, predominantly children, disproportionately affected despite children constituting only approximately 10% of VFR travelers [49]. For measles, ECDC data for 2023 document 2361 EU/EEA cases, predominantly in unvaccinated children under 5 years, driven in part by imported and import-linked transmission. By contrast, typhoid fever, malaria, and dengue show broadly consistent geographic and travel-purpose distributions across both the present synthesis and ECDC/GeoSentinel surveillance, providing partial internal validation of the patterns identified in this review. This comparison illustrates that the targeted disease scope of the present synthesis, while methodologically justified, means that conditions primarily documented through notification systems constitute a clinically significant but systematically absent component of the full epidemiological picture.

Seasonal trends may also influence-selected imported infections. For example, dengue among returned travelers has shown clear seasonal and annual variation, reflecting both travel patterns and transmission dynamics in destination regions [50]. In addition, seasonal patterns of vector activity and transmission in destination countries may further shape the timing of infections.

Another important epidemiological concern is the occurrence of diagnostic delays. Imported infections in pediatric populations may initially present with nonspecific symptoms, and clinicians in non-endemic European settings may have limited experience with certain tropical or travel-related diseases [4,8]. This can lead to delayed recognition and treatment, potentially increasing the risk of complications and secondary transmission.

Finally, gaps in pre-travel preparation, including vaccination, chemoprophylaxis, and access to travel medicine services, remain important contributing factors, particularly among VFR and migrant families [47,48,51,52,53]. Addressing these gaps requires targeted, multilevel public health strategies that go beyond generic educational messaging. Three specific policy interventions are supported by the evidence base of this review. First, the subsidization or full public funding of pre-travel vaccinations and malaria chemoprophylaxis for pediatric VFR travelers, recognizing that cost is a well-documented barrier to pre-travel care uptake in migrant and low-income families [47,48,51,52,53]. Second, the integration of travel health assessment into routine primary pediatric care for children from migrant families, rather than relying exclusively on specialist travel medicine clinics that may be geographically or financially inaccessible. Lastly, the development of culturally and linguistically adapted pre-travel health information targets families traveling to high-endemicity regions, delivered through community health workers, schools, and pediatric primary care settings. These interventions are consistent with recommendations from international travel medicine bodies [6,47,48] and would be most impactful if implemented as part of a coordinated European framework rather than fragmented national initiatives.

### 4.3. Why Data Are Limited

Current knowledge on the epidemiology of imported diseases in children and adolescents remains limited due to several structural and methodological barriers. A major challenge is the insufficient representation of the pediatric population in travel medicine research [54,55]. Children are often treated merely as a subgroup of adults, which prevents a reliable assessment of age-specific clinical risks [56]. This situation is further complicated by the fragmented epidemiological surveillance system in the European Union; differences in national reporting requirements and variable diagnostic capacity across laboratories result in pan-European data that are often incomplete and fragmented.

Another key challenge is the lack of standardized denominators, that is, precise information on the total number of traveling children who constitute the at-risk population. Without such data, accurate estimation of incidence rates as well as absolute risk estimation are not achievable through the present synthesis and remains difficult, limiting analyses to descriptive statistics of reported cases [57]. Interpretation is further complicated by inconsistent age stratification used across countries and studies. Discrepancies in defining the boundaries between early childhood and adolescence hinder the conduct of coherent meta-analyses and may obscure important epidemiological trends characteristic of specific stages of child development [56].

### 4.4. Comparison with Adult Data

Direct pediatric evidence on several aspects of travel-associated infections remains limited, and, in selected domains, interpretation is informed by indirect comparisons with adult traveler data. Large adult surveillance networks consistently demonstrate patterns that closely mirror those observed in pediatric cohorts, including the predominance of sub-Saharan Africa as the source of imported malaria, the central role of visiting-friends-and-relatives (VFR) travel, and the high burden of enteric and febrile illnesses among returning travelers [2,58,59,60]. Analyses from these networks indicate that VFR travelers are consistently overrepresented among reported cases of severe and preventable infections, largely due to the lower uptake of pre-travel advice and prophylaxis, delayed healthcare seeking, and prolonged or repeated exposure in endemic regions [2,5,59].

These observations are further supported by surveillance studies and clinical reviews of returned travelers, which consistently identify malaria, dengue, and enteric infections among major diagnostic considerations in post-travel illness [60,61,62]. Importantly, pediatric cohorts included in the present review reproduce these patterns, particularly with regard to the dominance of *Plasmodium falciparum* infections acquired in sub-Saharan Africa and the overrepresentation of VFR travel, as demonstrated across the included European studies [17,19,20,28].

While pediatric-specific datasets confirm many of these trends, important differences must be acknowledged. Children are typically embedded within family travel patterns and are therefore disproportionately represented among VFR travelers, which may amplify exposure risk compared to adult tourist populations [2,3,6]. In addition, pediatric cohorts often demonstrate different clinical presentations and healthcare utilization patterns, including a higher likelihood of hospital-based evaluation and diagnostic work-up [11,24,25]. However, the absence of standardized reporting across pediatric studies limits direct comparison with adult cohorts, and conclusions regarding relative frequency or severity should therefore be interpreted cautiously [2,59]. Overall, adult data provide a valuable contextual framework, but the present findings underscore the need for pediatric-specific surveillance and analysis to accurately characterize the epidemiology of imported infections in this population [3].

### 4.5. Clinical Implications

The findings of this review highlight several clinically relevant considerations for the management of children returning from international travel. First, a structured and systematic travel history remains essential in all pediatric patients presenting with febrile, gastrointestinal, or unexplained systemic symptoms, with particular attention to travel destination, timing, type of exposure, and purpose of travel, especially VFR status, which has consistently been associated with overrepresentation among reported cases of severe and preventable infections [2,5,47,48,59]. Second, the consistent overrepresentation of preventable infections, including malaria and enteric fever, underscores the critical importance of pre-travel counseling, vaccination, and chemoprophylaxis in pediatric populations, particularly among migrant families traveling to endemic regions, as emphasized in international guidelines [10,47,48].

Third, the observed heterogeneity and frequent incompleteness of reporting across studies point to the need for strengthened, standardized surveillance systems integrating clinical, microbiological, and epidemiological data, ideally harmonized across European settings and linked to existing international surveillance platforms such as GeoSentinel Surveillance Network and EuroTravNet [2,59]. Finally, while the present review provides a comprehensive overview of epidemiological patterns, the heterogeneity of available evidence and the predominance of observational data preclude the formulation of specific therapeutic recommendations, which should continue to rely on established clinical guidelines and pathogen-specific evidence [4,47].

### 4.6. Research Recommendations

Several key recommendations can be formulated to guide future epidemiological research regarding imported infectious diseases in the pediatric population. First and foremost, there is a critical need for methodological standardization and consistency in clinical data reporting. The current literature is characterized by significant heterogeneity, which hinders robust meta-analysis and the reliable comparison of results across different European countries. Future studies should, therefore, strictly adhere to uniform case definitions in accordance with the guidelines provided by the European Center for Disease Prevention and Control (ECDC) or the World Health Organization (WHO). Equally important is the implementation of precise age stratification to allow for the separate analysis of infants, school-aged children, and adolescents. Such an approach would enable the identification of specific exposure patterns and clinical courses, which differ depending on the biological and behavioral developmental stage of the young patient.

Another priority should be moving beyond single-center studies toward the establishment and strengthening of multicenter, pan-European registries. Expanding surveillance networks, such as EuroTravNet [59], by integrating smaller clinical facilities could improve the representativeness and statistical power of research samples. This is particularly vital for monitoring rare diseases and tracking emerging pathogens or antimicrobial resistance trends. Parallel to the development of clinical databases, a fundamental challenge remains the harmonization of surveillance denominators. Future research projects should not be limited to reporting the number of diagnosed cases but should strive to link these data with statistics regarding the total number of traveling children. Only such an integrated approach will allow for the accurate estimation of real incidence rates and a reliable assessment of the epidemiological patterns associated with travel to specific geographical regions.

The disproportionate burden of imported infectious diseases among pediatric VFR travelers identified in this review reflects a constellation of socioeconomic and cultural barriers that standard travel medicine frameworks have largely failed to address. These include financial barriers to pre-travel consultations and vaccines, which are not publicly funded in most European countries for healthy travelers, as well as lower health literacy regarding travel-related risks, cultural norms that normalize extended stays in endemic-region households without protective measures, and structural barriers to accessing specialist travel medicine services, including language difficulties, work schedule constraints, and geographic distance from travel clinics [6,47,48,51,52,53]. The consistent overrepresentation of VFR children in preventable infection categories, including malaria and typhoid fever, suggests that informational campaigns alone are insufficient. Evidence-based policy responses should include the following: (1) universal public funding of essential pre-travel vaccines and malaria chemoprophylaxis for children of VFR families, analogous to the publicly funded childhood immunization schedule; (2) mandatory travel health screening integration into school entry health checks and pediatric well-child visits for children with planned international travel; (3) dedicated outreach programs targeting migrant communities through culturally competent community health workers and peer educators; (4) harmonized European guidance on pre-travel care entitlements for pediatric VFR travelers, closing the current gap between national policies across EU member states. These recommendations are supported by evidence from both European and international travel medicine literature [6,47,48] and are proportionate to the burden of preventable morbidity documented in this review.

In the face of ongoing climate change and dynamic migration processes, the continuous monitoring of the evolving spectrum of imported diseases in children returning to Europe must become an integral component of health security strategies.

### 4.7. Strengths and Limitations

This systematic review has several methodological strengths. The study was prospectively registered in PROSPERO (CRD420251245531) and conducted in accordance with the PRISMA 2020 guidelines, ensuring transparency and reproducibility of the review process. The search strategy was comprehensive and covered three independent databases (PubMed, Scopus, and the Cochrane Library), with additional backward citation screening to enhance completeness. A strictly defined pediatric PICO framework was applied throughout the review, representing a deliberate methodological approach in a field where pediatric populations are frequently combined with adult cohorts or insufficiently stratified. Study selection and data extraction were performed independently by two reviewers, minimizing selection bias.

An additional strength is the use of study design-specific tools for risk-of-bias assessment, including the Newcastle–Ottawa Scale for cohort studies, an adapted Newcastle–Ottawa Scale for cross-sectional studies, and the Joanna Briggs Institute Critical Appraisal Checklists for case series and prevalence studies. This approach allowed for a more appropriate evaluation of methodological quality across heterogeneous study designs, rather than applying a single tool across fundamentally different types of evidence. Importantly, studies were not excluded solely on the basis of design category, which preserved the breadth of the available epidemiological data while allowing for quality-informed interpretation.

While the JBI Critical Appraisal Checklist for Studies Reporting Prevalence Data was not designed specifically for hospital-based case series or surveillance datasets, it was selected as the most operationally appropriate available instrument for studies that, while not reporting prevalence in a strict epidemiological sense, did report frequency data within defined clinical populations using explicit case definitions and analytical methods. The representativeness domain, which is the most problematic for hospital-based cohorts lacking identifiable sampling frames, was assessed with particular caution and was not used as a criterion for study exclusion. Given the descriptive, non-pooled nature of the synthesis, this limitation does not materially affect the principal findings. No validated appraisal tool specifically designed for imported infectious disease surveillance cohorts or post-travel clinical case series currently exists; the JBI Prevalence Checklist therefore represents a pragmatic and transparent methodological choice within the constraints of available instruments.

The review also employed a structured and conservative data aggregation strategy, including the strict inclusion of pediatric-specific numerical data, separation of syndromic and etiological diagnoses, and avoidance of redistribution of mixed infection categories. This approach reduced the risk of artificial inflation of pathogen frequencies and enhanced the interpretability of descriptive patterns.

Nevertheless, several limitations should be acknowledged. First, the majority of included studies were retrospective and hospital-based, which introduces a systematic ascertainment bias and the related spectrum effect [8]. Conditions requiring hospital admission, including malaria, typhoid fever, and invasive parasitic infections, are systematically overrepresented relative to self-limiting illnesses managed in primary or outpatient care, such as viral gastroenteritis and mild traveler’s diarrhea. The prominence of *P. falciparum* malaria in the aggregated pathogen dataset is therefore in part a function of this ascertainment structure rather than an accurate reflection of the true community burden of imported infections. The single available outpatient dataset, by Herbinger et al. [25], illustrates this contrast directly, with malaria accounting for fewer than 2% of post-travel diagnoses in that ambulatory setting. This spectrum bias cannot be resolved through secondary synthesis and represents a fundamental constraint on the interpretation of all pathogen-level frequency data in the present review.

Second, although a subset of malaria-focused studies shared a sufficiently common outcome, namely the proportion of *P. falciparum* among identified Plasmodium species, to permit formal heterogeneity assessment, no I^2^ statistic or Cochran Q test was calculated. This represents a methodological limitation: the narrative assessment of heterogeneity across malaria studies, while supported by the descriptive consistency of reported proportions, lacks the quantitative basis that formal heterogeneity statistics would have provided. Future syntheses of malaria-specific pediatric data from European settings would benefit from the formal pooling of species proportions using variance-stabilizing transformations.

Third, there is a fundamental asymmetry in the diagnostic certainty of reported pathogen categories. Malaria counts derive almost exclusively from parasitologically confirmed cases, whereas gastrointestinal diagnoses frequently combine microbiologically confirmed pathogens with syndromic clinical categories. Serological diagnoses, used for dengue, leishmaniasis, and schistosomiasis, may capture prior rather than active infection depending on antibody class and titre threshold applied, potentially overestimating active case counts. This heterogeneity in diagnostic certainty precludes the direct comparison of absolute case counts across pathogen groups. Readers seeking pathogen-specific diagnostic detail are directed to the Methods sections of the original studies cited in Supplementary Table S6.

Fourth, the risk-of-bias assessment used four design-specific instruments. For NOS-assessed studies, a score of ≥5 out of nine stars was considered indicative of acceptable methodological quality. For JBI-assessed studies, retention decisions were based on three core domains: clarity of case definition, adequacy of case ascertainment, and transparency of outcome reporting. All 31 retained studies demonstrated adequate performance on these core domains and post hoc sensitivity analysis restricted to higher-quality studies confirmed that the principal descriptive findings were not materially altered.

Fifth, the VFR category encompasses substantially heterogeneous subpopulations, including second-generation migrants visiting family abroad, first-generation returnees, newly arrived migrants, and children on employment-related family stays, that differ considerably in baseline immunity, exposure duration, and healthcare access. The internal variance within the VFR category may in some cohorts exceed the variance between VFR and tourist travelers, limiting the interpretive value of VFR as a unified epidemiological construct.

Sixth, the targeted rather than exhaustive disease scope of the search strategy means that certain clinically relevant travel-associated conditions are underrepresented or absent from the aggregated dataset. Hepatitis A and measles are the most clinically significant examples. While both meet the operational definition of an imported infection adopted in this review, they were not consistently represented in post-travel clinical cohort studies of the type eligible for inclusion, as their epidemiology is primarily captured through national notification registries and outbreak investigations rather than clinical cohort research. This reflects a structural feature of the available literature rather than an exclusion based on endemicity. Similarly, emerging or low-frequency arboviral conditions, including Oropouche virus infection, West Nile fever, and tick-borne encephalitis in the context of imported pediatric illness, were not systematically captured given their limited representation in the pediatric post-travel literature at the time of the search. As the epidemiology of imported arboviroses continues to evolve with expanding vector ranges and increasing travel volumes, future reviews should consider broader arboviral search strategies to avoid the systematic underrepresentation of emerging imported pathogens.

Seventh, the scope of the geographic analysis carries a definitional limitation. “Europe” was operationally defined as the European Union and its 27 current member states, as specified in the search strategy (Supplementary Table S1). Nevertheless, a small proportion of reported travel exposures could not be fully aligned with the conceptual definition of imported infection as infection acquired outside Europe. Review of Supplementary Table S6 identified 135 of 10,043 reported travel exposures (1.3%) classified under the Europe destination category. These exposures were contributed by six studies: Sondén et al. [22] (*n* = 7), Bird et al. [11] (approximately *n* = 14), Naudin et al. [24] (*n* = 67), Satarvandi et al. [36] (*n* = 8), Ria et al. [39] (*n* = 33), and Guery et al. [44] (*n* = 6). These cases were retained in the main descriptive analysis to preserve the original reporting structure of the included studies and to avoid arbitrary post hoc reclassification. In addition, Soriano–Arandes et al. [42] reported 10 exposures within a combined “Europe and North America” category. These cases were not included in the Europe-only count because they could not be reliably attributed to Europe alone and were therefore treated as a separate geographically ambiguous category. Sensitivity checks excluding Europe-classified exposures, and a stricter check excluding both Europe-classified exposures and the combined Europe/North America category, did not alter the principal geographic interpretation, as Africa and Asia remained the dominant reported exposure regions. These findings indicate that intra-European or geographically ambiguous exposures introduced only limited definitional imprecision and did not drive the main geographic conclusions of the review. A further limitation concerns the aggregation of exposures from the Americas/Caribbean in the main geographic figure. This category combines epidemiologically distinct regions, and infection risks for children returning from North America are not directly comparable to those for children returning from tropical or subtropical regions of Latin America, South America, or the Caribbean. Therefore, the Americas/Caribbean category should be interpreted only as a broad descriptive reporting category. More detailed subregional information, where available, is preserved in Supplementary Table S6.

Eighth, age-stratified analysis by developmental subgroup was not feasible, as none of the included studies provided systematically extractable age-disaggregated data across infants, school-aged children, and adolescents. This represents a fundamental gap that limits the characterization of age-specific epidemiological patterns.

Ninth, the heterogeneous temporal range of included studies, spanning data collection from the early 1990s to 2024, limits formal trend analysis, as standardized denominators and consistent case definitions across time periods were not available.

Tenth, a proportion of studies included mixed adult–pediatric populations without fully disaggregated pediatric data, leading to their partial exclusion from quantitative synthesis for specific variables, potentially underestimating the burden of certain infections.

Eleventh, reporting bias is likely, given the unequal distribution of surveillance systems, diagnostic capacity, and travel medicine expertise across European countries, resulting in the overrepresentation of data from Western and Southern Europe.

Taken together, these limitations highlight the need for cautious interpretation of aggregated findings and underscore the importance of standardized, pediatric-specific surveillance and reporting in future research.

## 5. Conclusions

Imported infectious diseases in children and adolescents returning to Europe encompass a clinically heterogeneous spectrum that extends well beyond malaria. Malaria, predominantly caused by *Plasmodium falciparum* acquired in sub-Saharan Africa, remains the most consistently reported infection in malaria-focused cohorts. However, gastrointestinal infections, enteric fever, dengue, leishmaniasis, and parasitic conditions represent a substantial share of post-travel morbidity in unselected pediatric populations. Children traveling as VFR are disproportionately represented across all infection categories, reflecting socioeconomic, cultural and structural barriers that standard pre-travel care frameworks have insufficiently addressed.

These findings have direct implications for clinical practice and public health. Pediatric clinicians in non-endemic European settings should routinely incorporate travel history, including destination, travel purpose, and VFR status, into the diagnostic evaluation of febrile or gastrointestinal illness following international travel. A malaria-only diagnostic approach is inadequate given the breadth of the documented spectrum. The consistently high burden among VFR children, much of it preventable, provides a strong rationale for publicly funded pre-travel vaccination and chemoprophylaxis for children from migrant families, an intervention that most European healthcare systems have yet to implement systematically.

The methodological heterogeneity of the available evidence limits direct cross-study comparability and precludes precise burden-of-disease estimation. Progress in this field will require harmonized case definitions, systematic age stratification and the integration of routine surveillance data with post-travel cohort studies, particularly for vaccine-preventable conditions that fall outside the scope of current clinical research designs.

## Data Availability

Data supporting this review are available within the article and Supplementary Materials.

## Supplementary Materials

The following supporting information can be downloaded at https://www.mdpi.com/article/10.3390/pathogens15060621/s1, Table S1: Search strategies used in PubMed, Scopus, and the Cochrane Library; Table S2: Results of individual study assessments done with Newcastle–Ottawa Scale for cross-sectional studies; Table S3: Results of quality assessment of the individual included studies using the Newcastle–Ottawa Scale for cohort studies; Table S4: Results of individual study assessments done with the Joanna Briggs Institute (JBI) Critical Appraisal Checklist for Case Series; Table S5: Results of individual study assessments done with the Joanna Briggs Institute (JBI) Critical Appraisal Checklist for Studies Reporting Prevalence Data; Table S6: Detailed study-level extracted pediatric case data included in the descriptive synthesis of imported infectious diseases among children and adolescents returning to Europe, stratified by infection type, travel destination, and reported reason for travel. Supplementary File S1 PRISMA 2020 Checklist [63]. Reference [63] is cited in the Supplementary Materials.

## Abbreviations

The following abbreviations are used in this manuscript:

AI	Artificial intelligence
ATB	Adventure travel and backpacking
CA	California
EAggEC	Enteroaggregative *Escherichia coli*
ECDC	European Center for Disease Prevention and Control
EIEC	Enteroinvasive *Escherichia coli*
EPEC	Enteropathogenic *Escherichia coli*
ETEC	Enterotoxigenic *Escherichia coli*
JBI	Joanna Briggs Institute
*L. aethiopica*	*Leishmania aethiopica*
*L. donovani*	*Leishmania donovani*
*L. major*	*Leishmania major*
*L. mexicana*	*Leishmania mexicana*
*L. tropica*	*Leishmania tropica*
*L.* (*Viannia*)	*Leishmania* (*Viannia*)
NA	Not available
NOS	Newcastle–Ottawa Scale
*P. falciparum*	*Plasmodium falciparum*
*P. malariae*	*Plasmodium malariae*
*P. ovale*	*Plasmodium ovale*
*P. vivax*	*Plasmodium vivax*
PICO	Population, Intervention/Exposure, Comparator, Outcome
PRISMA	Preferred Reporting Items for Systematic Reviews and Meta-Analyses
PROSPERO	International Prospective Register of Systematic Reviews
*S. haematobium*	*Schistosoma haematobium*
*S. intercalatum*	*Schistosoma intercalatum*
*S. mansoni*	*Schistosoma mansoni*
*S.* Paratyphi A	*Salmonella enterica* serovar Paratyphi A
*S.* Paratyphi B	*Salmonella enterica* serovar Paratyphi B
*S.* Typhi	*Salmonella enterica* serovar Typhi
STEC	Shiga toxin-producing *Escherichia coli*
UK	United Kingdom
USA	United States of America
VFR	Visiting friends and relatives
WHO	World Health Organization
