# Supplementary material for: Epidemiology and Spectrum of Imported Infectious Diseases in Children and Adolescents Returning to Europe: A Systematic Review
Source: Pathogens. 2026 Jun 9;15(6):621. doi: 10.3390/pathogens15060621 (PMC13306174; doi:10.3390/pathogens15060621)
Supplement: Supplementary file 1 [file pathogens-15-00621-s001.zip › pathogens-4341962-supplementary.pdf]

Table S1. Search strategies used in PubMed, Scopus, and the Cochrane Library.

**PubMed**
("Travel"[Mesh] OR travel[tiab] OR "Travel Medicine"[Mesh] OR "travel medicine"[tiab] OR "Tourism"[Mesh] OR tourism[tiab]) AND ("Communicable Diseases"[Mesh] OR "communicable disease*"[tiab] OR "infection*"[tiab] OR "infectious disease*"[tiab] OR "Travel-Related Illness"[Mesh] OR "travel-related illness"[tiab] OR "traveler's diarrhea"[tiab] OR "Zika Virus Infection"[Mesh] OR "zika virus infection"[tiab] OR "Zika Virus"[Mesh] OR "zika virus"[tiab] OR "zika fever"[tiab] OR Zika[tiab] OR "Leishmaniasis"[Mesh] OR leishmaniasis[tiab] OR "Leishmania"[Mesh] OR leishmania[tiab] OR "Paratyphoid Fever"[Mesh] OR "paratyphoid fever"[tiab] OR Paratyphoid[Mesh] OR paratyphoid[tiab] OR "Typhoid Fever"[Mesh] OR "typhoid fever"[tiab] OR Typhoid[Mesh] OR typhoid[tiab] OR "Chikungunya Fever"[Mesh] OR "chikungunya fever"[tiab] OR "Chikungunya Virus"[Mesh] OR "chikungunya virus"[tiab] OR Chikungunya[tiab] OR "Malaria"[Mesh] OR malaria[tiab] OR "Plasmodium"[Mesh] OR plasmodium[tiab] OR "Dengue"[Mesh] OR dengue[tiab] OR "Dengue Virus"[Mesh] OR "dengue virus"[tiab] OR "dengue fever"[tiab] OR "Schistosomiasis"[Mesh] OR schistosomiasis[tiab] OR "Schistosoma"[Mesh] OR schistosoma[tiab]) AND ("Pediatrics"[Mesh] OR pediatr*[tiab] OR "Child"[Mesh] OR child*[tiab] OR "Adolescent"[Mesh] OR adolescen*[tiab]) AND ("European Union"[Mesh] OR "European Union"[tiab] OR "Austria"[Mesh] OR Austria[tiab] OR "Belgium"[Mesh] OR Belgium[tiab] OR "Bulgaria"[Mesh] OR Bulgaria[tiab] OR "Croatia"[Mesh] OR Croatia[tiab] OR "Cyprus"[Mesh] OR Cyprus[tiab] OR "Czech Republic"[Mesh] OR "Czech Republic"[tiab] OR "Denmark"[Mesh] OR Denmark[tiab] OR "Estonia"[Mesh] OR Estonia[tiab] OR "Finland"[Mesh] OR Finland[tiab] OR "France"[Mesh] OR France[tiab] OR "Germany"[Mesh] OR Germany[tiab] OR "Greece"[Mesh] OR Greece[tiab] OR "Hungary"[Mesh] OR Hungary[tiab] OR "Ireland"[Mesh] OR Ireland[tiab] OR "Italy"[Mesh] OR Italy[tiab] OR "Latvia"[Mesh] OR Latvia[tiab] OR "Lithuania"[Mesh] OR Lithuania[tiab] OR "Luxembourg"[Mesh] OR Luxembourg[tiab] OR "Malta"[Mesh] OR Malta[tiab] OR "Netherlands"[Mesh] OR Netherlands[tiab] OR "Poland"[Mesh] OR Poland[tiab] OR "Portugal"[Mesh] OR Portugal[tiab] OR "Romania"[Mesh] OR Romania[tiab] OR "Slovakia"[Mesh] OR Slovakia[tiab] OR "Slovenia"[Mesh] OR Slovenia[tiab] OR "Spain"[Mesh] OR Spain[tiab] OR "Sweden"[Mesh] OR Sweden[tiab])
**Scopus**
(TITLE-ABS-KEY("travel") OR INDEXTERMS("travel") OR TITLE-ABS-KEY("travel medicine") OR INDEXTERMS("travel medicine") OR TITLE-ABS-KEY("tourism") OR INDEXTERMS("tourism")) AND (TITLE-ABS-KEY("communicable disease*") OR INDEXTERMS("communicable disease*") OR TITLE-ABS-KEY("infection*") OR INDEXTERMS("infection*") OR TITLE-ABS-KEY("infectious disease*") OR INDEXTERMS("infectious disease*") OR TITLE-ABS-KEY("travel-related illness") OR INDEXTERMS("travel-related illness") OR TITLE-ABS-KEY("traveler's diarrhea") OR INDEXTERMS("traveler's diarrhea") OR TITLE-ABS-KEY("zika virus infection") OR INDEXTERMS("zika virus infection") OR TITLE-ABS-KEY("zika virus") OR INDEXTERMS("zika virus") OR TITLE-ABS-KEY("zika fever") OR INDEXTERMS("zika fever") OR TITLE-ABS-KEY("zika") OR TITLE-ABS-KEY("leishmaniasis") OR INDEXTERMS("leishmaniasis") OR TITLE-ABS-KEY("leishmania") OR INDEXTERMS("leishmania") OR TITLE-ABS-KEY("paratyphoid fever") OR INDEXTERMS("paratyphoid fever") OR TITLE-ABS-KEY("paratyphoid") OR TITLE-ABS-KEY("typhoid fever") OR INDEXTERMS("typhoid fever") OR TITLE-ABS-KEY("typhoid") OR TITLE-ABS-KEY("chikungunya fever") OR INDEXTERMS("chikungunya fever") OR TITLE-ABS-KEY("chikungunya virus") OR INDEXTERMS("chikungunya virus") OR TITLE-ABS-KEY("chikungunya") OR TITLE-ABS-KEY("malaria") OR INDEXTERMS("malaria") OR TITLE-ABS-KEY("plasmodium") OR INDEXTERMS("plasmodium") OR TITLE-ABS-KEY("dengue") OR INDEXTERMS("dengue") OR TITLE-ABS-KEY("dengue virus") OR INDEXTERMS("dengue virus") OR TITLE-ABS-KEY("dengue fever") OR INDEXTERMS("dengue fever") OR TITLE-ABS-KEY("schistosomiasis") OR INDEXTERMS("schistosomiasis") OR TITLE-ABS-KEY("schistosoma") OR INDEXTERMS("schistosoma")) AND (TITLE-ABS-KEY("pediatr*") OR INDEXTERMS("pediatrics") OR TITLE-ABS-KEY("child*") OR INDEXTERMS("child") OR

TITLE-ABS-KEY("adolescen*") OR INDEXTERMS("adolescent")) AND (TITLE-ABS-KEY("european union") OR INDEXTERMS("european union") OR TITLE-ABS-KEY("austria") OR INDEXTERMS("austria") OR TITLE-ABS-KEY("belgium") OR INDEXTERMS("belgium") OR TITLE-ABS-KEY("bulgaria") OR INDEXTERMS("bulgaria") OR TITLE-ABS-KEY("croatia") OR INDEXTERMS("croatia") OR TITLE-ABS-KEY("cyprus") OR INDEXTERMS("cyprus") OR TITLE-ABS-KEY("czech republic") OR INDEXTERMS("czech republic") OR TITLE-ABS-KEY("denmark") OR INDEXTERMS("denmark") OR TITLE-ABS-KEY("estonia") OR INDEXTERMS("estonia") OR TITLE-ABS-KEY("finland") OR INDEXTERMS("finland") OR TITLE-ABS-KEY("france") OR INDEXTERMS("france") OR TITLE-ABS-KEY("germany") OR INDEXTERMS("germany") OR TITLE-ABS-KEY("greece") OR INDEXTERMS("greece") OR TITLE-ABS-KEY("hungary") OR INDEXTERMS("hungary") OR TITLE-ABS-KEY("ireland") OR INDEXTERMS("ireland") OR TITLE-ABS-KEY("italy") OR INDEXTERMS("italy") OR TITLE-ABS-KEY("latvia") OR INDEXTERMS("latvia") OR TITLE-ABS-KEY("lithuania") OR INDEXTERMS("lithuania") OR TITLE-ABS-KEY("luxembourg") OR INDEXTERMS("luxembourg") OR TITLE-ABS-KEY("malta") OR INDEXTERMS("malta") OR TITLE-ABS-KEY("netherlands") OR INDEXTERMS("netherlands") OR TITLE-ABS-KEY("poland") OR INDEXTERMS("poland") OR TITLE-ABS-KEY("portugal") OR INDEXTERMS("portugal") OR TITLE-ABS-KEY("romania") OR INDEXTERMS("romania") OR TITLE-ABS-KEY("slovakia") OR INDEXTERMS("slovakia") OR TITLE-ABS-KEY("slovenia") OR INDEXTERMS("slovenia") OR TITLE-ABS-KEY("spain") OR INDEXTERMS("spain") OR TITLE-ABS-KEY("sweden") OR INDEXTERMS("sweden"))

Cochrane Library

ID	Search	Hits
#1	MeSH descriptor: [Travel] explode all trees	423
#2	(travel):ti,ab,kw	5422
#3	MeSH descriptor: [Travel Medicine] explode all trees	5
#4	(travel medicine):ti,ab,kw	536
#5	MeSH descriptor: [Tourism] explode all trees	4
#6	(tourism):ti,ab,kw	83
#7	#1 OR #2 OR #3 OR #4 OR #5 OR #6	5512
#8	MeSH descriptor: [Communicable Diseases] explode all trees	31398
#9	(communicable disease*):ti,ab,kw	4809
#10	MeSH descriptor: [Infections] explode all trees	111197
#11	(infection*):ti,ab,kw	157156
#12	(infectious disease*):ti,ab,kw	10148
#13	MeSH descriptor: [Travel-Related Illness] explode all trees	51
#14	(travel-related illness):ti,ab,kw	19
#15	(traveler's diarrhea):ti,ab,kw	136
#16	MeSH descriptor: [Zika Virus Infection] explode all trees	69
#17	(zika virus infection):ti,ab,kw	118
#18	MeSH descriptor: [Zika Virus] explode all trees	38
#19	("Zika virus"):ti,ab,kw	132
#20	(zika fever):ti,ab,kw	72
#21	(zika):ti,ab,kw	171
#22	MeSH descriptor: [Leishmaniasis] explode all trees	557
#23	(leishmaniasis):ti,ab,kw	973
#24	MeSH descriptor: [Leishmania] explode all trees	153
#25	(leishmania):ti,ab,kw	383
#26	MeSH descriptor: [Paratyphoid Fever] explode all trees	37
#27	(paratyphoid fever):ti,ab,kw	229
#28	(paratyphoid):ti,ab,kw	278
#29	MeSH descriptor: [Typhoid Fever] explode all trees	362
#30	(typhoid fever):ti,ab,kw	701
#31	(typhoid):ti,ab,kw	876
#32	MeSH descriptor: [Chikungunya Fever] explode all trees	83
#33	(chikungunya fever):ti,ab,kw	144
#34	MeSH descriptor: [Chikungunya virus] explode all trees	29

#35	(chikungunya virus):ti,ab,kw	137
#36	(chikungunya):ti,ab,kw	211
#37	MeSH descriptor: [Malaria] explode all trees	4309
#38	(malaria):ti,ab,kw	7934
#39	MeSH descriptor: [Plasmodium] explode all trees	1284
#40	(plasmodium):ti,ab,kw	3453
#41	MeSH descriptor: [Dengue] explode all trees	490
#42	(dengue):ti,ab,kw	1025
#43	MeSH descriptor: [Dengue Virus] explode all trees	168
#44	(dengue virus):ti,ab,kw	486
#45	(dengue fever):ti,ab,kw	490
#46	MeSH descriptor: [Schistosomiasis] explode all trees	464
#47	(schistosomiasis):ti,ab,kw	718
#48	MeSH descriptor: [Schistosoma] explode all trees	208
#49	(schistosoma):ti,ab,kw	464
#50	#8 OR #9 OR #10 OR #11 OR #12 OR #13 OR #14 OR #15 OR #16 OR #17 OR #18 OR #19 OR #20 OR #21 OR #22 OR #23 OR #24 OR #25 OR #26 OR #27 OR #28 OR #29 OR #30 OR #31 OR #32 OR #33 OR #34 OR #35 OR #36 OR #37 OR #38 OR #39 OR #40 OR #41 OR #42 OR #43 OR #44 #45 OR #46 OR #47 OR #48 OR #49	210851
#51	MeSH descriptor: [Pediatrics] explode all trees	1075
#52	(pediatr*):ti,ab,kw	44909
#53	MeSH descriptor: [Child] explode all trees	84008
#54	(child*):ti,ab,kw	222758
#55	MeSH descriptor: [Adolescent] explode all trees	139384
#56	(adolescen*):ti,ab,kw	181054
#57	#51 OR #52 OR #53 OR #54 OR #55 OR #56	347510
#58	MeSH descriptor: [European Union] explode all trees	116
#59	(european union):ti,ab,kw	1681
#60	MeSH descriptor: [Austria] explode all trees	515
#61	(austria):ti,ab,kw	2611
#62	MeSH descriptor: [Belgium] explode all trees	834
#63	(belgium):ti,ab,kw	3233
#64	MeSH descriptor: [Bulgaria] explode all trees	51
#65	(bulgaria):ti,ab,kw	275
#66	MeSH descriptor: [Croatia] explode all trees	106
#67	(croatia):ti,ab,kw	314
#68	MeSH descriptor: [Cyprus] explode all trees	31
#69	(cyprus):ti,ab,kw	155
#70	MeSH descriptor: [Czech Republic] explode all trees	190
#71	(czech republic):ti,ab,kw	916
#72	MeSH descriptor: [Denmark] explode all trees	2605
#73	(denmark):ti,ab,kw	7118
#74	MeSH descriptor: [Estonia] explode all trees	46
#75	(estonia):ti,ab,kw	206
#76	MeSH descriptor: [Finland] explode all trees	1564
#77	(finland):ti,ab,kw	3486
#78	MeSH descriptor: [France] explode all trees	2591
#79	(france):ti,ab,kw	9423
#80	MeSH descriptor: [Germany] explode all trees	4613
#81	(germany):ti,ab,kw	16912
#82	MeSH descriptor: [Greece] explode all trees	465
#83	(greece):ti,ab,kw	1526
#84	MeSH descriptor: [Hungary] explode all trees	185
#85	(hungary):ti,ab,kw	855
#86	MeSH descriptor: [Ireland] explode all trees	467
#87	(ireland):ti,ab,kw	3058
#88	MeSH descriptor: [Italy] explode all trees	2937

#89 (italy):ti,ab,kw 9165
#90 MeSH descriptor: [Latvia] explode all trees 30
#91 (latvia):ti,ab,kw 113
#92 MeSH descriptor: [Lithuania] explode all trees 59
#93 (lithuania):ti,ab,kw 203
#94 MeSH descriptor: [Luxembourg] explode all trees 15
#95 (luxembourg):ti,ab,kw 77
#96 MeSH descriptor: [Malta] explode all trees 12
#97 (malta):ti,ab,kw 57
#98 MeSH descriptor: [Netherlands] explode all trees 4870
#99 (netherlands):ti,ab,kw 12416
#100 MeSH descriptor: [Poland] explode all trees 657
#101 (poland):ti,ab,kw 2009
#102 MeSH descriptor: [Portugal] explode all trees 258
#103 (portugal):ti,ab,kw 1127
#104 MeSH descriptor: [Romania] explode all trees 152
#105 (romania):ti,ab,kw 507
#106 MeSH descriptor: [Slovakia] explode all trees 38
#107 (slovakia):ti,ab,kw 160
#108 MeSH descriptor: [Slovenia] explode all trees 65
#109 (slovenia):ti,ab,kw 317
#110 MeSH descriptor: [Spain] explode all trees 2373
#111 (spain):ti,ab,kw 7982
#112 MeSH descriptor: [Sweden] explode all trees3217
#113 (sweden):ti,ab,kw 8896
#114 #58 OR #59 OR #60 OR #61 OR #61 OR #62 OR #63 OR #64 OR #65 OR #66 OR #67 OR #68 OR #69 OR #70 OR #71 OR #72 OR #73 OR #74 OR #75 OR #76 OR #77 OR #78 OR #79 OR #80 OR #81 OR #82 OR #83 OR #84 OR #85 OR #86 OR #87 OR #88 OR #89 OR #90 OR #91 OR #92 OR #93 OR #94 OR #95 OR #96 OR #97 OR #98 OR #99 OR #100 OR #101 OR #102 OR #103 OR #104 OR #105 OR #106 OR #107 OR #108 OR #109 OR #110 OR #111 OR #112 OR #113 81967
#115 #7 AND #50 AND #57 AND #114 28

Table S2. Results of individual study assessments done with Newcastle–Ottawa Scale for cross-sectional studies.

Ref.	Selection				Comparability	Outcome		TOTAL
	Representativeness of the sample	Sample size	Non-responders	Asceratinment of exposure	Based on design and analysis	Assessment of outcome	Statistical test	
Navarro et al.(2024)	*			*		**	*	5 points (Satisfactory studies)
Bird et al. (2024)	*			**		*	*	5 points (Satisfactory studies)
Herbinger et al. (2012)	*			*	**	*	*	6 (Satisfactory studies)
Satarvandi et al. (2024)	*			*	**	**	*	7 points (Good studies)

The Newcastle–Ottawa Scale for cross-sectional studies uses a "star-based system" to evaluate three key domains: selection, comparability, and outcome. Studies can receive up to five stars for selection, three for outcome, and two for comparability. Overall, each study is assigned a methodological quality score ranging from 0 to 10 stars, where a higher number of stars reflects better methodological quality.

Table S3. Results of quality assessment of the individual included studies using the Newcastle-Ottawa Scale for cohort studies.

**Ref.**	**Selection**				**Comparability**	**Outcome**			**TOTAL**
	Representativeness of the exposed cohort	Selection of the non-exposed cohort	Ascertainment of Exposure	Demonstration that outcome of interest was not present at the start of the study	Comparability of cohorts on the basis of the design or analysis	Assessment of Outcome	Was follow-up long enough for outcomes to occur	Adequacy of follow up of cohorts	
Luise et al. (2017)	*	*	*			*	*	*	6points(Poor quality)
Soriano-Arandes et al.(2016	*	*	*		*	*	*		6points(Good quality)
Sondén et al. (2025)		*	*	*	*	*	*	*	7 points (Good quality)
Zanotti et al. (2017)	*	*	*		*	*	*		6 points(Good quality)
Mornand et al. (2017)	*	*	*		**	*	*		7points(Good quality
Dubos et al. (2010)	*	*	*		*	*	*	*	7 points(Good quality)
(28) Ladhani et al. (2010)	*	*	*		**	*	*	*	8points(Good quality)

Good quality: 3 or 4 stars in selection domain AND 1 or 2 stars in comparability domain AND 2 or 3 stars in outcome/exposure domain; Fair quality: 2 stars in selection domain AND 1 or 2 stars in comparability domain

AND 2 or 3 stars in outcome/exposure domain; Poor quality: 0 or 1 star in selection domain OR 0 stars in comparability domain OR 0 or 1 stars in outcome/exposure domain

Table S4. Results of individual study assessments done with the Joanna Briggs Institute (JBI) Critical Appraisal Checklist for Case Series.

Ref.	Q1	Q2	Q3	Q4	Q5	Q6	Q7	Q8	Q9	Q10	Total yes (max 10)
Ria et al. (2024)	Y	Y	Y	Y	Y	Y	Y	Y	UC	Y	9
Selimaj Kontoni et al. (2023)	Y	Y	Y	Y	Y	Y	Y	Y	Y	Y	10
Finale et al. (2020)	Y	Y	Y	Y	Y	N	N	N	N	N	5
Pommelet et al. (2018)	Y	Y	Y	Y	Y	Y	Y	Y	Y	Y	10
Mellado-Sola et al. (2025)	UC	N	N	Y	Y	Y	Y	Y	Y	Y	7
Agagliati et al. (2022)	Y	UC	UC	Y	Y	Y	Y	Y	Y	Y	8
Torres-Fernandez et al. (2021)	Y	Y	N	N	N	Y	Y	Y	Y	Y	7
Mendoza-Palomar et al. (2020)	Y	N	N	Y	Y	Y	Y	Y	Y	Y	8
Soto Sánchez et al. (2016)	Y	Y	Y	Y	Y	Y	Y	Y	Y	Y	10
Maltezou et al. (2013)	Y	Y	Y	Y	N	Y	Y	Y	Y	Y	9
Naudin et al. (2012)	Y	Y	N	Y	N	Y	Y	Y	Y	Y	8
Garcia-Villarrubia et al. (2011)	Y	Y	Y	Y	Y	Y	Y	Y	Y	UC	9
Arnáez et al. (2010)	Y	Y	Y	Y	Y	Y	Y	Y	Y	Y	10
Guery et al. (2021)	Y	Y	Y	N	N	Y	Y	Y	Y	Y	8
Cnops et al. (2020)	Y	Y	Y	UC	Y	Y	Y	Y	Y	Y	9
Minodier et. al. (2011)	Y	Y	Y	UC	UC	Y	Y	Y	Y	Y	8

Y- Yes, N- No, UC-Unclear

Q1-Were there clear criteria for inclusion in the case series?

Q2-Was the condition measured in a standard, reliable way for all participants included in the case series?

Q3-Were valid methods used for identification of the condition for all participants included in the case series?

Q4-Did the case series have consecutive inclusion of participants?

Q5-Did the case series have complete inclusion of participants?

Q6-Was there clear reporting of the demographics of the participants in the study?

Q7-Was there clear reporting of clinical information of the participants?

Q8-Were the outcomes or follow up results of cases clearly reported?

Q9-Was there clear reporting of the presenting site(s)/clinic(s) demographic information?

Q10-Was statistical analysis appropriate?

Table S5. Results of individual study assessments done with the Joanna Briggs Institute (JBI) Critical Appraisal Checklist for Studies Reporting Prevalence Data.

**Ref.**	**Q1**	**Q2**	**Q3**	**Q4**	**Q5**	**Q6**	**Q7**	**Q8**	**Q9**	**Total yes (max 9)**
Enkelmann et al. (2025)	Y	Y	Y	Y	Y	Y	Y	Y	NA	8
Pouletty et al. (2018)	N	Y	N	Y	N	Y	Y	N	N	4
Söbirk et al. (2018)	N	Y	Y	Y	N	UC	N	Y	N/A	4
Vygen-Bonnet and Stark (2018)	Y	Y	Y	Y	Y	Y	Y	Y	NA	8

Y-Yes, N-No, UC-Unclear, N/A- Not applicable

Q1-Was the sample frame appropriate to address the target population?

Q2-Were study participants sampled in an appropriate way?

Q3-Was the sample size adequate?

Q4-Were the study subjects and the setting described in detail?

Q5-Was the data analysis conducted with sufficient coverage of the identified sample?

Q6-Were valid methods used for the identification of the condition?

Q7-Was the condition measured in a standard, reliable way for all participants?

Q8-Was there appropriate statistical analysis?

Q9-Was the response rate adequate, and if not, was the low response rate managed appropriately?

Table S6. Detailed study-level extracted pediatric case data included in the descriptive synthesis of imported infectious diseases among children and adolescents returning to Europe, stratified by infection type, travel destination, and reported reason for travel.

	Selimaj Kontoni et al.	Finale et al. [1]	Luise et al.	Agagliati et al.	Zanotti et al.	Mornand et al. [2]	Soto Sánchez et al. [3]	Vygen-Bonnet and Stark	Maltezou et al.	Garcia-Villarrubia et al.	Arnáez et al.	Dubos et al. [4]	Ladhani et al.	Minodier et al. [5]	Sondén et al.	Badillo Navarro et al. [6]	Bird et al. [7]	Torres-Fernandez et al. [8]	Herbinger et al. [9]	Naudin et al.	Satarvandi et al.	Enkelmann et al.	Ria et al. [10]	Pouletty et al.	Söbirk et al.	Pommelet et al. [11]	Soriano-Arandes et al.	Mellado-Sola et al.	Mendoza-Palomar et al.	Guery et al.	Cnops et al.	Sum
**INFECTION TYPE**																																
P. falciparum	144	150	46	68	170	4150	135	NA	7	121	43	~75	NA	95	NA	23	36	34	NA	NA	NA	0	0	0	0	0	0	0	0	0	0	5297
P. vivax	10	3	0	1	45	0	0	NA	12	25	1	~6	NA	1	NA	0	7	0	NA	NA	NA	0	0	0	0	0	0	0	0	0	0	111
P. malariae	1	0	0	0	1	0	3	NA	0	8	0	0	NA	0	NA	0	2	2	NA	NA	NA	0	0	0	0	0	0	0	0	0	0	17
P. ovale	3	8	2	2	9	0	10	NA	0	10	6	~3	NA	3	NA	0	1	0	NA	NA	NA	0	0	0	0	0	0	0	0	0	0	57
Mixed plasmodium infection	2	3	0	0	0	0	9	NA	2	5	5	~5	NA	4	NA	1	1	3	NA	NA	NA	0	0	0	0	0	0	0	0	0	0	40
Unknown/Not specified plasmodium species	0	9	0	1	0	0	8	NA	0	5	5	~49	NA	0	14	0	13	0	15	51	12	0	0	0	0	0	0	0	0	0	0	182
Malaria total	160	172	48	72	225	4150	147	NA	21	174	60	133	NA	95	14	24	60	39	15	51	12	0	0	0	0	0	0	0	0	0	0	5672
Dengue	0	0	0	0	0	0	0	0	0	0	0	0	0	0	5	2	4	0	18	0	4	0	0	0	0	0	0	46	0	0	0	79
S. typhi	0	0	0	0	0	0	0	0	0	0	0	0	0	0	0	0	0	0	0	0	0	356	0	0	0	44	0	0	0	0	0	400
Typhoid fever unspecified	0	0	0	0	0	0	0	0	0	0	0	0	0	0	3	0	21	3	0	0	3	0	0	0	0	0	0	0	0	0	0	30
S. paratyphi A	0	0	0	0	0	0	0	0	0	0	0	0	0	0	0	0	0	0	0	0	0	69	0	0	0	0	0	0	0	0	0	69
S. paratyphi B	0	0	0	0	0	0	0	0	0	0	0	0	0	0	0	0	0	0	0	0	0	180	0	0	0	0	0	0	0	0	0	180
S. paratyphi unspecified	0	0	0	0	0	0	0	0	0	0	0	0	0	0	0	0	0	0	0	0	0	0	0	0	0	6	0	0	0	0	0	6

Salmonella enteritis	0	0	0	0	0	0	0	0	0	0	0	0	0	0	0	0	0	0	27	0	0	0	0	0	0	0	0	0	0	0	0	27
Salmonella unspecified	0	0	0	0	0	0	0	0	0	0	0	0	0	0	0	0	0	0	0	0	0	0	0	16	0	0	0	0	0	0	0	16
EAggEC	0	0	0	0	0	0	0	0	0	0	0	0	0	0	0	0	0	0	0	0	0	0	0	32	0	0	0	0	0	0	0	32
EPEC	0	0	0	0	0	0	0	0	0	0	0	0	0	0	0	0	0	0	0	0	0	0	0	26	0	0	0	0	0	0	0	26
ETEC	0	0	0	0	0	0	0	0	0	0	0	0	0	0	0	0	0	0	0	0	0	0	0	19	0	0	0	0	0	0	0	19
EIEC/Shigella	0	0	0	0	0	0	0	0	0	0	0	0	0	0	0	0	0	0	0	0	0	0	0	16	0	0	0	0	0	0	0	16
STEC	0	0	0	0	0	0	0	0	0	0	0	0	0	0	0	0	0	0	0	0	0	0	12	4	0	0	0	0	0	0	0	16
Shigella enteritis	0	0	0	0	0	0	0	0	0	0	0	0	0	0	0	0	0	0	11	0	0	0	0	0	0	0	0	0	0	0	0	11
Gastroenteritis [12]	0	0	0	0	0	0	0	0	0	0	0	0	0	0	37	0	178	22	0	0	34	0	0	0	0	0	0	0	0	0	0	271
Acute diarrhoea unspecified	0	0	0	0	0	0	0	0	0	0	0	0	0	0	0	0	0	0	0	146	0	0	0	0	0	0	0	0	0	0	0	146
Dysentery	0	0	0	0	0	0	0	0	0	0	0	0	0	0	0	0	12	0	0	0	0	0	0	0	0	0	0	0	0	0	0	12
Campylobacter	0	0	0	0	0	0	0	0	0	0	0	0	0	0	0	0	0	0	29	0	0	0	0	10	0	0	0	0	0	0	0	39
Clostridium difficile	0	0	0	0	0	0	0	0	0	0	0	0	0	0	0	0	0	0	0	0	0	0	0	3	0	0	0	0	0	0	0	3
Sapovirus	0	0	0	0	0	0	0	0	0	0	0	0	0	0	0	0	0	0	0	0	0	0	0	11	0	0	0	0	0	0	0	11
Norovirus	0	0	0	0	0	0	0	0	0	0	0	0	0	0	0	0	0	0	0	0	0	0	0	10	0	0	0	0	0	0	0	10
Rotavirus	0	0	0	0	0	0	0	0	0	0	0	0	0	0	0	0	0	0	0	0	0	0	0	9	0	0	0	0	0	0	0	9
Astrovirus	0	0	0	0	0	0	0	0	0	0	0	0	0	0	0	0	0	0	0	0	0	0	0	4	0	0	0	0	0	0	0	4
Adenovirus	0	0	0	0	0	0	0	0	0	0	0	0	0	0	0	0	0	0	0	0	0	0	0	2	0	0	0	0	0	0	0	2
Cryptosporidium	0	0	0	0	0	0	0	0	0	0	0	0	0	0	0	0	0	0	0	0	0	0	0	11	0	0	0	0	0	0	0	11
Giardiasis	0	0	0	0	0	0	0	0	0	0	0	0	0	0	0	0	0	0	62	0	0	0	0	8	0	0	61	0	0	0	0	131
Leishmaniasis	0	0	0	0	0	0	0	0	0	0	0	0	0	0	0	0	1	0	0	0	0	0	0	0	58	0	0	0	0	106 (25 immigrants)	0	165
Protozoan	0	0	0	0	0	0	0	0	0	0	0	0	0	0	0	0	0	0	0	0	0	0	0	0	0	0	142	0	0	0	0	142

Helminthes	0	0	0	0	0	0	0	0	0	0	0	0	0	0	0	0	0	0	0	0	0	0	0	0	0	0	97	0	0	0	0	97
Leptospirosis	0	0	0	0	0	0	0	0	0	0	0	0	0	0	1	0	0	0	0	0	0	0	0	0	0	0	0	0	0	0	0	1
Chikungunya	0	0	0	0	0	0	0	0	0	0	0	0	0	0	0	1	0	0	0	0	0	0	0	0	0	0	0	0	0	0	0	1
Amebiasis	0	0	0	0	0	0	0	0	0	0	0	0	0	0	0	1	0	0	19	0	0	0	0	0	0	0	0	0	0	0	0	20
Cutaneous larva migrans	0	0	0	0	0	0	0	0	0	0	0	0	0	0	0	0	0	0	24	0	0	0	0	0	0	0	0	0	0	0	0	24
Schistosomiasis	0	0	0	0	0	0	0	0	0	0	0	0	0	0	0	0	0	0	32	0	0	0	0	0	0	0	0	0	51 (43 immigrants)	0	18	101
**TRAVEL DESTINATION**																																
Africa overall	154	168	48	71	185	4136	144	NA	8	146	59	107	159	~94	108	88	471	103	359	399	90	NA	10	55	NA	25	206	1	51	62	18	7525
Sub-saharan Africa	154	21	0	0	185	~372	144	NA	1	0	57	0	0	0	108	87	471	103	0	185	90	NA	0	29	NA	14	184	1	0	0	0	2206
North Africa	0	0	0	0	0	0	0	NA	0	0	0	0	0	0	0	1	0	0	0	214	0	NA	10	25	NA	11	22	0	0	44	0	327
South Africa	0	0	0	0	0	0	0	NA	1	0	0	0	0	0	0	0	0	0	0	0	0	NA	0	1	NA	0	0	0	0	0	18	20
West Africa	0	143	0	0	0	~2110	0	NA	4	0	2	86	0	~5	0	0	0	0	0	0	0	NA	0	0	NA	0	0	0	0	18	0	2368
East Africa	0	4	0	0	0	~827	0	NA	2	0	0	21	0	~89	0	0	0	0	0	0	0	NA	0	0	NA	0	0	0	0	0	0	943
Africa, region not specified	0	0	48	71	0	~827	0	NA	0	146	0	0	159	0	0	0	0	0	359	0	0	NA	0	0	NA	0	0	0	51	0	0	1661
Asia overall	6	3	0	1	38	8	3	NA	8	15	0	3	0	0	0	0	622	9	269	22	0	NA	0	4	NA	14	170	0	0	2	0	1197
Central Asia	0	0	0	0	0	0	0	NA	0	0	0	0	0	0	0	0	0	0	0	16	0	NA	0	0	NA	0	0	0	0	0	0	16
South Asia	0	0	0	0	0	0	0	NA	0	0	0	0	0	0	0	0	622	0	0	0	0	NA	0	0	NA	0	0	0	0	2	0	624
Southeast Asia	0	0	0	0	0	0	0	NA	0	0	0	0	0	0	0	0	0	0	0	6	0	NA	0	0	NA	0	0	0	0	0	0	6
Indian subcontinent	0	3	0	1	38	0	0	NA	0	0	0	0	0	0	0	0	0	0	0	0	0	NA	0	0	NA	14	0	0	0	0	0	56
Indian Subcontinent and Asia	0	0	0	0	0	0	0	NA	0	0	0	0	0	0	0	0	0	0	0	0	0	NA	0	0	NA	0	170	0	0	0	0	170
Pakistan/ India/ Afghanistan	0	0	0	0	0	0	0	NA	8	0	0	0	0	0	0	0	0	0	0	0	0	NA	0	0	NA	0	0	0	0	0	0	8

Asia, region not specified	6	0	0	0	0	8	3	NA	0	15	0	3	0	0	0	0	0	9	269	0	0	NA	0	4	NA	0	0	0	0	0	0	317
Americas overall	0	0	0	0	0	5	0	NA	0	3	1	0	0	~1	17	12	~42	56	146	24	11	NA	0	0	NA	1	220	25	0	6	0	570
North America	0	0	0	0	0	0	0	NA	0	0	0	0	0	0	2	0	0	0	0	2	1	NA	0	0	NA	0	0	0	0	4	0	9
South America	0	0	0	0	0	0	0	NA	0	0	0	0	0	~1	0	3	~42	56	0	0	0	NA	0	0	NA	1	0	8	0	2	0	113
Latin America	0	0	0	0	0	2	0	NA	0	3	1	0	0	0	0	1	0	0	146	0	10	NA	0	0	NA	0	0	0	0	0	0	163
Central and South America	0	0	0	0	0	0	0	NA	0	0	0	0	0	0	15	0	0	0	0	4	0	NA	0	0	NA	0	0	0	0	0	0	19
Caribbean	0	0	0	0	0	3	0	NA	0	0	0	0	0	0	0	8	0	0	0	0	0	NA	0	0	NA	0	0	17	0	0	0	28
West Indies	0	0	0	0	0	0	0	NA	0	0	0	0	0	0	0	0	0	0	0	18	0	NA	0	0	NA	0	0	0	0	0	0	18
Latin America and Caribbean	0	0	0	0	0	0	0	NA	0	0	0	0	0	0	0	0	0	0	0	0	0	NA	0	0	NA	0	220	0	0	0	0	220
America, region not specified	0	0	0	0	0	0	0	NA	0	0	0	0	0	0	0	0	0	0	0	0	0	NA	0	0	NA	0	0	0	0	0	0	0
Europe	0	0	0	0	0	0	0	NA	0	0	0	0	0	0	7	0	~14	0	0	67	8	NA	33	0	NA	0	0	0	0	6	0	135
Other	0	0	0	0	0	1	0	NA	0	0	0	0	0	0	0	0	0	0	0	26	0	NA	0	0	NA	1	0	0	0	30	0	58
Middle East	0	0	0	0	0	0	0	NA	0	0	0	0	0	0	0	0	0	0	0	24	0	NA	0	0	NA	1	0	0	0	30	0	55
Russia	0	0	0	0	0	0	0	NA	0	0	0	0	0	0	0	0	0	0	0	1	0	NA	0	0	NA	0	0	0	0	0	0	1
Pacific	0	0	0	0	0	1	0	NA	0	0	0	0	0	0	0	0	0	0	0	0	0	NA	0	0	NA	0	0	0	0	0	0	1
Oceania	0	0	0	0	0	0	0	NA	0	0	0	0	0	0	0	0	0	0	0	1	0	NA	0	0	NA	0	0	0	0	0	0	1
Mixed regions	0	0	0	0	0	0	0	NA	0	0	0	0	0	0	112	0	~56	0	0	0	93	NA	0	0	NA	0	10	7	0	0	0	278
Central and South American countries, Southeast and Central Asia	0	0	0	0	0	0	0	NA	0	0	0	0	0	0	0	0	0	0	0	0	0	NA	0	0	NA	0	0	7	0	0	0	7
Asia & Pacific	0	0	0	0	0	0	0	NA	0	0	0	0	0	0	84	0	0	0	0	0	66	NA	0	0	NA	0	0	0	0	0	0	150
East and Southeast Asia, Oceania	0	0	0	0	0	0	0	NA	0	0	0	0	0	0	0	0	~56	0	0	0	0	NA	0	0	NA	0	0	0	0	0	0	56

North Africa & Middle East	0	0	0	0	0	0	0	NA	0	0	0	0	0	0	28	0	0	0	0	0	27	NA	0	0	NA	0	0	0	0	0	0	55
Europe and North America	0	0	0	0	0	0	0	NA	0	0	0	0	0	0	0	0	0	0	0	0	0	NA	0	0	NA	0	10	0	0	0	0	10
Unknown/ not specified	0	1	0	0	2	0	0	NA	5	10	0	10	13	0	0	0	~209	20	0	0	0	NA	0	0	NA	0	0	13	0	0	0	283
**REASON FOR TRAVEL**																																
Tourism [13]	2	20	3	0	0	NA	0	NA	1	0	0	NA	117	NA	58	9	NA	4	400	474	NA	NA	NA	24	NA	2	34	7	4	15	18	1192
VFR [14]	109	0	42	53	173	NA	66	NA	6	108	14	NA	0	NA	39	43	NA	106	228	0	NA	NA	NA	35	NA	29	173	26	4	60	0	1314
Immigration [15]	0	26	3	10	45	NA	0	NA	7	143	46	NA	0	NA	0	45	NA	0	32	0	NA	NA	NA	0	NA	0	399	11	43	0	0	810
Migrant / Visitor [16]	49	0	0	2	0	NA	0	NA	0	0	0	NA	0	NA	18	0	NA	61	0	64	NA	NA	NA	0	NA	0	0	0	0	25	0	219
Work-related travel [17]	0	2	0	0	0	NA	0	NA	1	0	0	NA	0	NA	0	0	NA	0	50	0	NA	NA	NA	0	NA	0	0	0	0	0	0	53
Residence / Return of origin country	0	105	0	0	0	NA	0	NA	0	0	0	NA	0	NA	0	0	NA	0	0	0	NA	NA	NA	0	NA	0	0	0	0	0	0	105
Adoption	0	0	0	4	0	NA	0	NA	0	0	0	NA	0	NA	0	0	NA	0	0	0	NA	NA	NA	0	NA	0	0	0	0	0	0	4
Exchange program	0	0	0	0	0	NA	0	NA	0	0	0	NA	0	NA	0	0	NA	0	17	0	NA	NA	NA	0	NA	0	0	0	0	0	0	17
Missionary / volunteer	0	0	0	0	0	NA	0	NA	0	0	0	NA	0	NA	0	0	NA	0	23	0	NA	NA	NA	0	NA	0	0	0	0	0	0	23
Other / unknown [18]	0	19	0	3	6	NA	0	NA	1	0	0	NA	0	NA	0	3	NA	0	0	0	NA	NA	NA	0	NA	3	0	2	0	6	0	43

Data was pooled only for pediatric population and only when separation from adult population was feasible.

NA - not available (data not available or not reported)

1) Finale et al.: The paper reports 172 pediatric malaria cases overall, but the plasmodium species breakdown sums up to 173 cases, suggesting a probable reporting or typographical error in the results section. Therefore, 172 cases were counted.

2) Mornand et al.: Division into regions within Africa was estimated based on percentage values. Number of cases was not reported directly.

3) Soto Sánchez et al.: Mixed plasmodium infections were double counted within individual plasmodium species, information about overlapping species was not available. Final summary took into account the overall reported number of pediatric malaria cases.

4) Dubos et al.: The paper reports only percentages, therefore the number of cases was estimated based on the available data. Mixed infections were double counted within individual plasmodium species, information about overlapping species was not available. Final summary took into account the overall reported number of pediatric malaria cases.

5) Minodier et al.: 95 children were diagnosed with P. falciparum and 4 of them were coinfected: 3 patients with P. ovale and 1 patient with P. vivax. Division into continents and regions was estimated based on the percentage values as the number of cases was not reported directly.

6) Badillo Navarro et al.: Mixed plasmodium infection due to P. falciparum/P. vivax coinfection.

7) Bird et al.: Mixed plasmodium infection due to P. vivax-P. ovale. Division into travel destinations was estimated based on the percentage values as the number of cases was not reported directly (except for South Asia and Sub-Saharan Africa).

8) Torres-Fernandez et al.: mixed plasmodium infections due to P. falciparum-P. malariae, P. falciparum-P. vivax, and P. falciparum-P. ovale.

9) Herbinger et al.: Travel information was available for 774 travelers with German origin

10) Ria et al.: Country of infection ≠ travel destination. Country of infection was included in travel destination.

11) Pommelet et al.: Travel information available only for 41 cases and reason for travel for 34 cases

12) includes bacterial, viral, parasitic and unspecified gastroenteritis

13) Tourism includes: ATB- Adventure travel and backpacking including other tourist travels with low hygienic standard, Package tour- Package tour including other tourist travels with high hygienic standard, travelers, tourists

14) VFR includes: VFR traveler, VFR immigrant, VFR born abroad

15) Immigration includes: Immigrants, Immigration / immigrant

16) Migrant/Visitor includes: Migrants, Visitors / newly arrived migrants

17) Work-related travel includes: Parent/guardian working abroad, Business trip

18) Other / unknown includes: 1 case of repatriation to Greece and 1 expatriate (worker, missionary)
