# Supplementary material for: Epidemiology and Spectrum of Imported Infectious Diseases in Children and Adolescents Returning to Europe: A Systematic Review
Source: Pathogens. 2026 Jun 9;15(6):621. doi: 10.3390/pathogens15060621 (PMC13306174; doi:10.3390/pathogens15060621)
Supplement: Supplementary file 1 [file pathogens-15-00621-s001.zip › Supplementary File S1 PRISMA 2020 Checklist.pdf]

PRISMA 2020 Checklist

Section and Topic	Item #	Checklist item	Location where item is reported
**TITLE**			
Title	1	Identify the report as a systematic review.	Title
**ABSTRACT**			
Abstract	2	See the PRISMA 2020 for Abstracts checklist.	Separate checklist
**INTRODUCTION**			
Rationale	3	Describe the rationale for the review in the context of existing knowledge.	Section 1 (Introduction)
Objectives	4	Provide an explicit statement of the objective(s) or question(s) the review addresses.	Section 1, final paragraph; PICO framework stated in Abbreviations
**METHODS**			
Eligibility criteria	5	Specify the inclusion and exclusion criteria for the review and how studies were grouped for the syntheses.	Sections 2.3–2.4
Information sources	6	Specify all databases, registers, websites, organisations, reference lists and other sources searched or consulted to identify studies. Specify the date when each source was last searched or consulted.	Section 2.5; PubMed, Scopus, Cochrane Library; last search: 26 December 2025
Search strategy	7	Present the full search strategies for all databases, registers and websites, including any filters and limits used.	Supplementary Table S1 (full search strategies for all three databases)
Selection process	8	Specify the methods used to decide whether a study met the inclusion criteria of the review, including how many reviewers screened each record and each report retrieved, whether they worked independently, and if applicable, details of automation tools used in the process.	Section 2.6 (two independent reviewers; Rayyan platform; third arbitrator for discrepancies)
Data collection process	9	Specify the methods used to collect data from reports, including how many reviewers collected data from each report, whether they worked independently, any processes for obtaining or confirming data from study investigators, and if applicable, details of automation tools used in the process.	Section 2.7; Supplementary Table S6 (standardised extraction form; two independent reviewers)
Data items	10a	List and define all outcomes for which data were sought. Specify whether all results that were compatible with each outcome domain in each study were sought (e.g. for all measures, time points, analyses), and if not, the methods used to decide which results to collect.	Section 2.7 (all outcome domains listed and defined)
	10b	List and define all other variables for which data were sought (e.g. participant and intervention characteristics, funding sources). Describe any assumptions made about any missing or unclear information.	Sections 2.7–2.9 (participant characteristics, travel purpose, region; harmonisation rules defined)
Study risk of bias assessment	11	Specify the methods used to assess risk of bias in the included studies, including details of the tool(s) used, how many reviewers assessed each study and whether they worked independently, and if applicable, details of automation tools used in the process.	Section 2.10 (NOS cohort, NOS cross-sectional, JBI Case Series, JBI Prevalence; three independent reviewers); Supplementary Tables S2–S5
Effect measures	12	Specify for each outcome the effect measure(s) (e.g. risk ratio, mean difference) used in the synthesis or presentation of results.	Section 2.12 (absolute counts and proportions used as effect measures throughout)
Synthesis methods	13a	Describe the processes used to decide which studies were eligible for each synthesis (e.g. tabulating the study intervention characteristics and comparing against the planned groups for each synthesis (item #5)).	Sections 2.3, 2.8–2.9 (studies grouped by infection type, travel region and

Section and Topic	Item #	Checklist item	Location where item is reported
			travel purpose)
	13b	Describe any methods required to prepare the data for presentation or synthesis, such as handling of missing summary statistics, or data conversions.	Section 2.9 (harmonisation rules for age groups, pathogen categories and travel purposes defined)
	13c	Describe any methods used to tabulate or visually display results of individual studies and syntheses.	Sections 2.9, 2.12; Tables 2–4; Figures 2–4; Supplementary Table S6
	13d	Describe any methods used to synthesize results and provide a rationale for the choice(s). If meta-analysis was performed, describe the model(s), method(s) to identify the presence and extent of statistical heterogeneity, and software package(s) used.	Section 2.12 (descriptive synthesis selected; rationale provided based on high methodological heterogeneity precluding meta-analysis)
	13e	Describe any methods used to explore possible causes of heterogeneity among study results (e.g. subgroup analysis, meta-regression).	Section 3.7 (sources of heterogeneity explored across study design, setting, population and case definitions)
	13f	Describe any sensitivity analyses conducted to assess robustness of the synthesized results.	Section 2.12 (robustness of descriptive findings assessed through stratified sub-group comparisons)
Reporting bias assessment	14	Describe any methods used to assess risk of bias due to missing results in a synthesis (arising from reporting biases).	Section 2.12 (risk of bias due to missing results evaluated; publication bias assessed across included studies)
Certainty assessment	15	Describe any methods used to assess certainty (or confidence) in the body of evidence for an outcome.	Section 2.12; Section 3.9 (certainty of the body of evidence assessed in the context of study quality and heterogeneity)
**RESULTS**			
Study selection	16a	Describe the results of the search and selection process, from the number of records identified in the search to the number of studies included in the review, ideally using a flow diagram.	Section 3.1; Figure 1 (PRISMA 2020 flow diagram: 1558 records identified → 37 studies included)
	16b	Cite studies that might appear to meet the inclusion criteria, but which were excluded, and explain why they were excluded.	Section 3.1 (reasons for exclusion of full-text articles specified)
Study characteristics	17	Cite each included study and present its characteristics.	Section 3.2; Tables 2–4 (full characteristics of all 31 included studies)
Risk of bias in studies	18	Present assessments of risk of bias for each included study.	Section 3.9; Supplementary Tables S2–S5 (risk of bias assessed for each included study)
Results of individual studies	19	For all outcomes, present, for each study: (a) summary statistics for each group (where appropriate) and (b) an effect estimate and its precision (e.g. confidence/credible interval), ideally using structured tables or plots.	Tables 2–4; Supplementary Table S6 (study-level data presented for all analytical dimensions)

PRISMA

PRISMA 2020 Checklist

Section and Topic	Item #	Checklist item	Location where item is reported
Results of syntheses	20a	For each synthesis, briefly summarise the characteristics and risk of bias among contributing studies.	Section 3.9; Supplementary Tables S2–S5 (risk of bias summarised per contributing study)
	20b	Present results of all statistical syntheses conducted. If meta-analysis was done, present for each the summary estimate and its precision (e.g. confidence/credible interval) and measures of statistical heterogeneity. If comparing groups, describe the direction of the effect.	Sections 3.3–3.8; Figures 2–4 (counts and proportions presented per study and per synthesis)
	20c	Present results of all investigations of possible causes of heterogeneity among study results.	Section 3.7 (heterogeneity investigated across study design, population, geographic setting and case definitions)
	20d	Present results of all sensitivity analyses conducted to assess the robustness of the synthesized results.	Sections 2.12, 3.7 (robustness of synthesised findings evaluated across study subgroups)
Reporting biases	21	Present assessments of risk of bias due to missing results (arising from reporting biases) for each synthesis assessed.	Sections 2.12, 3.7 (risk of bias due to missing results evaluated for each synthesis domain)
Certainty of evidence	22	Present assessments of certainty (or confidence) in the body of evidence for each outcome assessed.	Sections 3.7, 3.9, 4.7 (certainty of evidence assessed per outcome domain)
**DISCUSSION**			
Discussion	23a	Provide a general interpretation of the results in the context of other evidence.	Sections 4.1–4.2 (results interpreted in the context of existing European and global travel medicine literature)
	23b	Discuss any limitations of the evidence included in the review.	Section 4.7 – Strengths and Limitations (limitations of included evidence detailed)
	23c	Discuss any limitations of the review processes used.	Section 4.7 – Strengths and Limitations (methodological limitations of the review process specified)
	23d	Discuss implications of the results for practice, policy, and future research.	Sections 4.5-4.6 and also Section 5 (clinical, public health and research implications stated)
**OTHER INFORMATION**			
Registration and protocol	24a	Provide registration information for the review, including register name and registration number, or state that the review was not registered.	Section 2.1; Abstract (PROSPERO, registration number CRD420251245531)
	24b	Indicate where the review protocol can be accessed, or state that a protocol was not prepared.	Section 2.1; protocol accessible via

PRISMA 2020 Checklist

Section and Topic	Item #	Checklist item	Location where item is reported
			PROSPERO (https://www.crd.york.ac.uk/prospero/)
	24c	Describe and explain any amendments to information provided at registration or in the protocol.	Section 2.1 (no amendments made to the registered protocol)
Support	25	Describe sources of financial or non-financial support for the review, and the role of the funders or sponsors in the review.	Funding section (no external funding received; authors declare independent conduct)
Competing interests	26	Declare any competing interests of review authors.	Conflicts of Interest section (no competing interests declared by any author)
Availability of data, code and other materials	27	Report which of the following are publicly available and where they can be found: template data collection forms; data extracted from included studies; data used for all analyses; analytic code; any other materials used in the review.	Data Availability Statement; Supplementary Tables S1–S6 (search strategies, extraction forms, risk-of-bias tables, study-level data)

From: Page MJ, McKenzie JE, Bossuyt PM, Boutron I, Hoffmann TC, Mulrow CD, et al. The PRISMA 2020 statement: an updated guideline for reporting systematic reviews. BMJ 2021;372:n71. doi: 10.1136/bmj.n71. This work is licensed under CC BY 4.0. To view a copy of this license, visit https://creativecommons.org/licenses/by/4.0/ Data Availability Statement; Supplementary Tables S1–S6
